# Synthesis, Molecular Docking, and Bioactivity Study of Novel Hybrid Benzimidazole Urea Derivatives: A Promising α-Amylase and α-Glucosidase Inhibitor Candidate with Antioxidant Activity

**DOI:** 10.3390/pharmaceutics15020457

**Published:** 2023-01-30

**Authors:** Lotfi M. Aroua, Abdulelah H. Alosaimi, Fahad M. Alminderej, Sabri Messaoudi, Hamdoon A. Mohammed, Suliman A. Almahmoud, Sridevi Chigurupati, Abuzar E. A. E. Albadri, Nejib H. Mekni

**Affiliations:** 1 Department of Chemistry, College of Science, Qassim University, Qassim Main Campus, King Abdulaziz Road, P.O. Box 6644, Al-Malida, Buraydah 51452, Saudi Arabia; 2 Laboratory of Structural Organic Chemistry—Synthesis and Physicochemical Studies (LR99ES14), Department of Chemistry, Faculty of Sciences of Tunis, University of Tunis El Manar, Tunis 2092, Tunisia; 3 Faculty of Sciences of Bizerte, Carthage University, Jarzouna, Bizerte 7021, Tunisia; 4 Department of Medicinal Chemistry and Pharmacognosy, College of Pharmacy, Qassim University, Buraidah 51452, Saudi Arabia; 5 Department of Pharmacognosy and Medicinal Plants, Faculty of Pharmacy, Al-Azhar University, Cairo 11371, Egypt; 6 Department of Biotechnology, Saveetha School of Engineering, Saveetha Institute of Medical and Technical Sciences, Saveetha University, Saveetha Nagar, Thandalam, Chennai 602105, India; 7 High Institute of Medical Technologies of Tunis, El Manar University, Tunis 1006, Tunisia

**Keywords:** benzimidazole-ureas, isocyanates, antioxidant, enzyme inhibition, diabetes mellitus, molecular docking simulation

## Abstract

A novel series of benzimidazole ureas **3a–h** were elaborated using 2-(1*H*-benzoimidazol-2-yl) aniline **1** and the appropriate isocyanates **2a–h**. The antioxidant and possible antidiabetic activities of the target benzimidazole-ureas **3a–h** were evaluated. Almost all compounds **3a–h** displayed strong to moderate antioxidant activities. When tested using the three antioxidant techniques, TAC, FRAP, and MCA, compounds **3b** and **3c** exhibited marked activity. The most active antioxidant compound in this family was compound **3g**, which had excellent activity using four different methods: TAC, FRAP, DPPH-SA, and MCA. In vitro antidiabetic assays against α-amylase and α-glucosidase enzymes revealed that the majority of the compounds tested had good to moderate activity. The most favorable results were obtained with compounds **3c**, **3e**, and **3g**, and analysis revealed that compounds **3c** (IC_50_ = 18.65 ± 0.23 μM), **3e** (IC_50_ = 20.7 ± 0.06 μM), and **3g** (IC_50_ = 22.33 ± 0.12 μM) had good α-amylase inhibitory potential comparable to standard acarbose (IC_50_ = 14.21 ± 0.06 μM). Furthermore, the inhibitory effect of **3c** (IC_50_ = 17.47 ± 0.03 μM), **3e** (IC_50_ = 21.97 ± 0.19 μM), and **3g** (IC_50_ = 23.01 ± 0.12 μM) on α-glucosidase was also comparable to acarbose (IC_50_ = 15.41 ± 0.32 μM). According to in silico molecular docking studies, compounds **3a–h** had considerable affinity for the active sites of human lysosomal acid α-glucosidase (HLAG) and pancreatic α-amylase (HPA), indicating that the majority of the examined compounds had potential anti-hyperglycemic action.

## 1. Introduction

Diabetes mellitus (DM) is a broad and complex endocrine disease, which is a major global health issue. It is a disease characterized by marked multiple organ dysfunction and failure caused by hyperglycemia resulting from insulin deficiency or dysregulation of the insulin action pathway [1].

Type 2 diabetes is brought on by a confluence of genetic factors linked to impaired insulin secretion and insulin resistance, as well as environmental factors such as obesity, overeating, a lack of exercise, stress, and aging. However, type 1 diabetes is the result of an autoimmune reaction to proteins of the islets cells of the pancreas [2]. Since 2019, an estimated 463 million people (8.8% of the adult population) have been diagnosed with diabetes; type II diabetes represents about 85% to 95% of all diabetes cases in developed countries, with an even higher percentage in developing nations [3,4].

**Figure 1:**
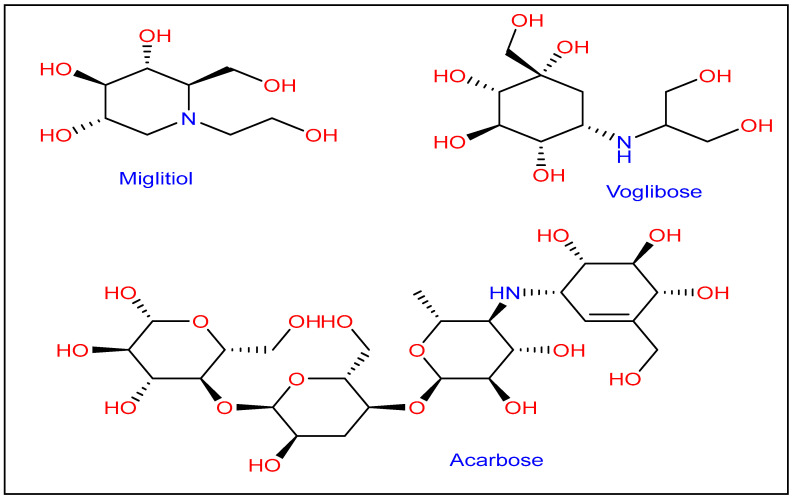
Type 2 diabetes mellitus drug on the market (T2DM).

α-Amylase is a calcium-based metalloenzyme that degrades saccharides into maltose by cleaving α (1–4) glycosidic bonds. It is abundant in human pancreatic juice and saliva, as well as in the saliva of some other mammals [5]. The small intestine contains some α-glucosidases, such as the hexamer-based hydrolyze enzyme, which converts polysaccharides and disaccharides into glucose by processing the non-reducing terminal 1,4-linked α-glucose residues. In diabetes, the inhibition of these two enzymes becomes very important as it reduces the digestion rate of carbohydrates, resulting in less glucose absorption in the bloodstream [6]. Three drugs are commercially available for diabetes: naturally derived glycosidase inhibitor molecules (acarbose, miglitol, and voglibose), as well as many other types of drugs [7] (Figure 1).

There appears to be a strong direct correlation between antioxidant and anti-diabetic effects; this relationship may be attributed to the significant role of oxidative stress in hyperglycemia and overall diabetic complications [8]. The evaluated levels of blood glucose are combined with the overproduction of free radicals “reactive oxygen species” [8]. In addition, part of the immunogenic damage to beta cells of the pancreas is mediated by ROS [9]. Therefore, natural and synthetic antioxidant compounds, e.g., phenolics and flavonoids, are promising candidates for the protection, treatment, and management of diabetic complications [10,11].

Furthermore, free radical scavengers and chelators have been reported for their potential role in diabetes management, such as reducing the vascular relaxation and neurovascular impairments associated with hyperalgesia, improving endoneurial blood flow, and overall reducing the production of OH- and the initiation of lipid peroxidation associated with vascular reactivity impairment and nerve function in diabetes [12].

Benzimidazole species have an excellent structural range that covers large biological and therapeutic application fields. The research describing the importance of structures containing benzimidazole cores has been extensively developed and possesses a wide range of bioactive activities, including antifungal [13], antitubercular [14,15], antiulcer and antimicrobial [16], antileishmanial [17], antiglycation and antioxidant [18], antimycobacterial [19], anti-HIV [20], antitumor [21], antiproliferative [22], anti-inflammatory [23,24], antiviral [25], and antihelminthic activities [26]. Several candidates of this class were tested for anti-tumor properties and showed activities against several tumor cell lines, including breast cancer, human cells, and human lung cancer cells [27,28,29,30,31,32,33].

In addition, some benzimidazole derivatives are well recognized for their antidiabetic potential, especially as potent α-glucosidase and α-amylase inhibitors. A multitude of benzimidazole derivatives were reviewed as α-amylase inhibitors including benzimidazole bearing sulfonamide [34], 2-arylbenzimidazole [35], 2-mercaptobenzimidazole [36], 2-aminobenzimidazole derivatives [37], substituted benzimidazole analogs [38], 2-(2-methyl-5-nitro-1*H*-imidazol-1-yl)ethylaryl ether [39], and benzimidazole-based Cu (II)/Zn (II) complexes [40].

Moreover, some benzimidazole structures having 5-bromo-2-arylbenzimidazole [41], conjugated biphenylbenzimidazole [42], benzimidazole linked with morpholine or piperazine [43], oxadiazole bearing benzimidazole motif [44], substituted arylbenzimidazol at para-position bridged with thiazolidinone [45], benzimidazole linked with Schiff base [46], benzimidazole bearing triazole [47], hybrid benzimidazole quinolinyl oxadiazole squeleton [48], hybrid benzimidazole with thiourea [49], and fluoro-2-substitued-1*H*-benzimidazol substituted with morpholine [50] were reported as α-glucosidase inhibitors. In this context, benzimidazole derived from 5-oxo-pyrido triazepine and aminomethyloxo-pyrimido [51], benzoylaryl benzimidazole [52], benzimidazol with substituted benzylidene containing 2,4-thiazolidinedione, diethyl malonate, and methylacetoacetate [53], and benzimidazole bridged with triazole [54] also exhibited excellent α-amylase and α-glucosidase activities.

The urea structure involves one of the most significant intermediates in organic and medicinal chemistry. This edifice is useful to develop biological active compounds, including anticancer, antiviral, antibacterial, and antihypertensive agents, which are clinically approved therapeutics [55,56,57,58]. Due to its remarkable structure, urea can stably form hydrogen bonds with target proteins and receptors. Furthermore, a targeted urea scaffold was used to find antidiabetic agents covering hybrid-urea pyrimidine-triazole [59], thiadiazole-urea bearing quinazolinone [60], sulfonylamino pyridinylbenzothiazol-urea [61], pyrimidine hybrid phenylaminourea [62], diarylurea containing quinazoline [63], urea moiety with coumarin-chalcone derivatives [64], and diarylurea linked with benzothiophene [65].

**Figure 2:**
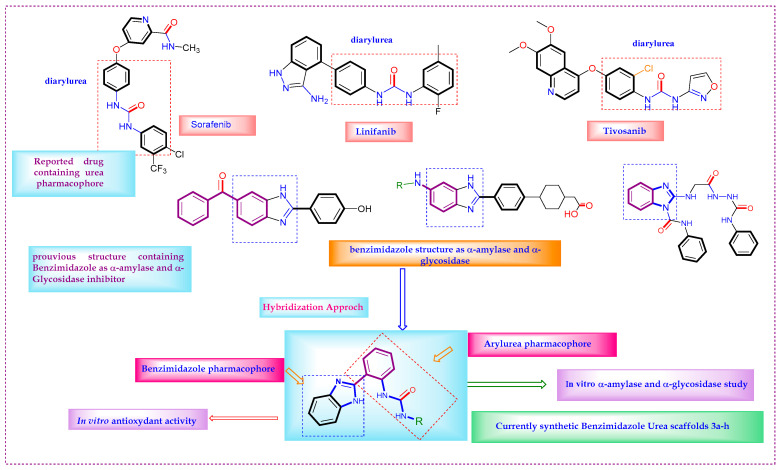
Design of novel hybrid benzimidazole connected with urea.

Following the previous scientific report, we elaborated herein a new target structure, containing both benzimidazole and diarylurea moieties in one component. Our purpose was to combine their properties, which represent a new route to improve the effectiveness of bioactive molecules (Figure 2).

There is a critical need to find better drugs to treat type I and type II diabetes. The discovery of new α-glucosidase and α-amylase inhibitor agents is an interesting method of obtaining such drugs. To contribute to the advancement of the design and the discovery of new bioactive species [66,67,68,69,70,71], we combined the two patterns, benzimidazole and urea, in the same molecule, allowing for potential antidiabetic agents, and having good anti-oxidant activity.

A simple route to novel hybrid benzimidazole urea compounds **3a-h** was utilized from the reaction of 2-(1*H*-benzimizadol-2-yl) aniline **1** and appropriate isocyanates substrates **2a-h**. The evaluation of antioxidant, α-amylase, and α-glucosidase activities was assessed. To estimate the interaction with α-amylase and α-glucosidase enzymes, an in silico molecular docking study of benzimidazole-urea was performed.

## 2. Results and Discussion

### 2.1. Chemistry

**Scheme 1:**
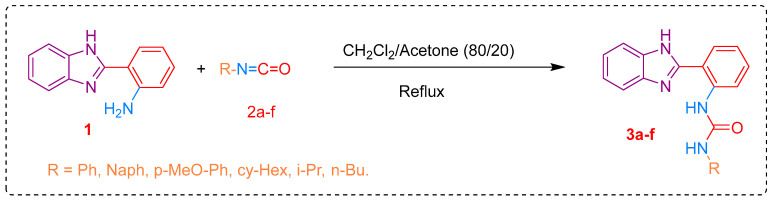
Synthesis of benzimidazole-urea **3a–f** from 2-(1*H*-benzoimidazol-2-yl)aniline **1** and isocyanates **2a–f**.

The synthesis of target benzimidazole-ureas **3a-f** was realized from commercially available 2-(1*H*-benzoimidazol-2-yl)aniline **1** and six different isocyanates **2a-f**. In a first attempt, the reaction of 2-(1*H*-benzoimidazol-2-yl)aniline **1** and naphthylisocyanate **2b** in anhydrous acetone produced the expected benzimidazole-urea **3b** with a moderate yield of 60% (Scheme 1).

To enhance the performance of this synthetic method, different reaction conditions were studied. The principal reaction parameters, including the solvent and number of equivalent reagents, were varied using the same substrates (1 and 2b). Among the investigated solvents: tetrahydrofuran, acetone, dichloromethane, and mixtures of dichloromethane/acetone, this latter mixture, in the respective proportions of 80/20, gave the best results with total substrate conversion and a 90% reaction yield.

As a result of optimizing the equivalent number of the substrate **2b** suitable for the reaction process, the substrate was fully converted with 1.5 equivalents of the isocyanate **2b**.

**Scheme 2:**
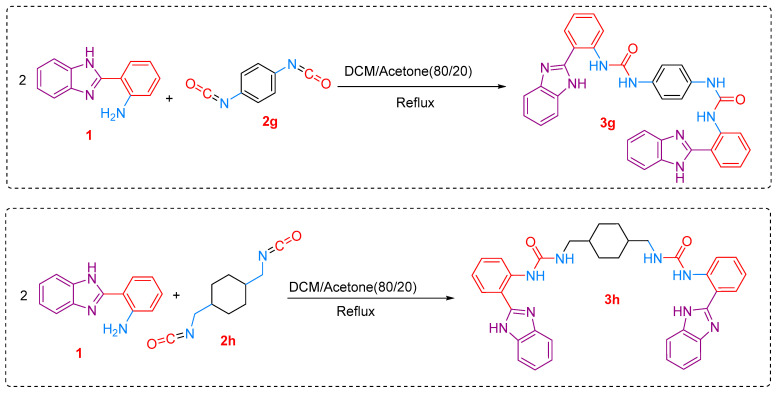
Synthesis of compounds **3g** and **3h** from 2-(1*H*-benzoimidazol-2-yl)aniline **1**.

1,4-phenelenediisocyanate **2g** and 1,4-bis(isocyanatomethyl)cyclohexane **2h** reacted with two equivalents of substrate **1** to afford the corresponding products **3g** and **3h** in good yields, 93% and 85%, respectively (Scheme 2).

The reactions progressed easily, offering good yields of the proposed substrates **3a-h**, and the results are collected in Table 1. It is worth noting that the reaction yields of the aryl substrates were higher than those of the alkyl and cycloalkyl ones. The low reaction yields as well as the rates of the reactions observed with alkyl and cycloalkyl isocyanate substrates may be due principally to the low reactivity of such radicals, which exert an inductive effect that decreases the isocyanate carbon electrophilicity.

The structure of novel prepared benzimidazole urea was determined by spectroscopic data from ^1^H and ^13^C NMR, FT-IR, and HRMS. The IR spectra of compound **3a** showed the absorption at 3249 cm^−1^ related to the NH group of the benzimidazole ring. The spectra also displayed stretching at 1672, 1586, and 1536 cm^−1^ assigned to (C=O), (C=N), and (C=C), respectively.

The ^1^H NMR spectra of compound **3a** showed a band at 13.08 ppm attributed to the NH group of benzimidazole. The amino proton of the urea group resonated at 12.02 ppm. Aromatic protons resonated in the range of 9.69–7.01 ppm. The compound **3a** was identified with ^13^C NMR. The spectra exhibited a singlet at 153.2 ppm corresponding to the carbon atom of the urea carbonyl group. In addition, the carbon of the benzimidazole ring appeared at 151.45 ppm. The high-resolution mass spectra of compound **3a** revealed the presence of a molecular peak with *m*/*z:* 328.12280 attributed to [M]^+^ compatible with the formula for C_20_H_16_N_4_O (see Supplementary Materials).

### 2.2. Biology

#### 2.2.1. Antioxidant Activity

Four in vitro assays were used to determine the antioxidant activity of the compounds **3a-h**. The methods used provide information about the compounds’ reducing capacity (TAC and FRAP methods), scavenging capability (DPPH-SA), and metal chelating activity (MCA). Table 2 gives the antioxidant levels for the compounds, measured in a comparable manner using three different in vitro mechanisms, namely, reducing, scavenging, and metal chelating activities.

In the series of benzimidazole ureas **3a-h**, compound **3g** demonstrated the highest reducing power for molybdenum (VI) with 10.06 ± 1.51 mM trolox equivalents in the total antioxidant capacity (TAC). The results of reducing activity also revealed that some compounds reduced molybdenum (VI) ions selectively in the TAC. For instance, compounds **3b**, **3c**, and **3f** showed significant molybdenum (VI) reducing activity compared to other compounds at levels of 5.16 ± 0.69, 4.43 ± 0.53, and 8.50 ± 0.87 mM trolox equivalents.

Regarding Table 2, the results of the ferric reducing antioxidant power (FRAP) of compounds **3a-h** indicated higher activities of derivatives **3a** and **3c** with 9.25 ± 0.20 and 8.65 ± 0.29 mM trolox equivalents, respectively. The most potent compound **3g** yielded the best results, with 16.12 ± 0.29 mM trolox equivalents.

The results of DPPH-SA of hybrid benzimidazole urea revealed the potency of derivatives **3d** and **3g** as free radical scavengers. Compound **3d** presented good activity with 2.89 ± 1.07 mM trolox equivalents. Compound **3g** also gave an excellent result with 3.86 ± 0.04 mM trolox equivalents. The other compounds exerted moderate DPPH-SA activity at values ranging from 1.70 to 0.96 mM trolox equivalents.

The metal chelating activity (MCA) of compounds **3a-h** manifested potential activity for the compound **3h** at the MCA level of 2.95 ± 0.21 mM EDTA equivalents due to the presence of two urea groups. In addition, compounds **3a-c** exhibited moderate MCA in the range of 1.36–1.62 mM EDTA equivalents.

The overall antioxidant activities of compounds **3a-h** confirmed that the majority manifested excellent to moderate activities. Benzimidazole-urea **3b** and **3c** displayed good activity with the three-antioxidant methods TAC, FRAP, and MCA. Targeted compound **3g** represented the most derivatives in this family, manifesting excellent activity in three methods: TAC, FRAP, and DPPH-SA. It is worth noting that compound **3g** also had significant activity for the TAC method, with 10.06 ± 1.51 mM trolox equivalents. Compound **3g** also gave higher activity with 16.12 ± 0.29 mM trolox equivalents for the FRAP method. Concerning the DPPH-SA method, compound **3g** also gave the best results with 3.86 ± 0.04 mM trolox equivalents.

#### 2.2.2. α-Amylase and α-Glucosidase Inhibition Activity

All the synthesized novel hybrid benzimidazole urea compounds **3a-h** were subjected for the evaluation of their possible α-amylase and α-glucosidase inhibitory activities. It is worth noting that the majority of the analogs were found to have good to moderately active IC_50_ values ranging from 18.65 ± 0.23 to 28.33 ± 0.02 µM for α-amylase inhibition and with the IC_50_ values ranging from 17.47 ± 0.03 to 29.01 ± 0.12 µM for α-glucosidase inhibition except compounds **3a**, **3b**, and **3h**. The inhibitory activities of α-amylase and α-glucosidase, according to Table 3, revealed that the tested compounds in the current study demonstrate higher activities than those reported in previous studies [47,72].

Consulting Table 3, the results indicate that compounds **3c** (IC_50_ = 18.65 ± 0.23 µM), **3e** (IC_50_ = 20.7 ± 0.06 µM), and **3g** (IC_50_ = 22.33 ± 0.12 µM) manifested a good α-amylase inhibitory potential comparable to standard acarbose (IC_50_ = 14.21 ± 0.06 µM) (Table 3). The same observation was mentioned with compounds **3c** (IC_50_ = 17.47 ± 0.03 µM), **3e** (IC_50_ = 21.97 ± 0.19 µM), and **3g** (IC_50_ = 23.01 ± 0.12 µM), which exerted an α-glucosidase inhibitory potential comparable also to standard drug acarbose (IC_50_ = 15.41 ± 0.32 µM).

**Figure 3:**
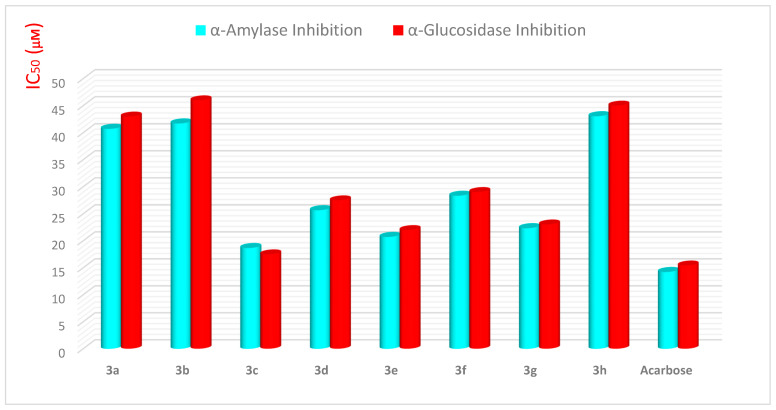
Benzimidazole-ureas **3a-h** inhibition activity against α-amylase and α-glucosidase.

As illustrated in Figure 3, compounds **3d** and **3f** displayed a moderate α-amylase inhibitory potential with IC_50_ = 25.65 ± 0.03 and IC_50_ = 28.33 ± 0.02 µM, respectively. The same results were mentioned with **3d** analogs with IC_50_ = 27.47 ± 0.13 and **3f** with IC_50_ = 29.01 ± 0.12 µM for α-glycosidase inhibition.

Indeed, the whole molecule played an important role in the inhibitory potential; however, limited SAR was rationalized by emphasizing the effects of varying structural features such as different substitutions on nitrogen of the amide side chain of urea, on the inhibitory potential in terms of IC_50_ values.

Comparing the inhibitory potential of benzimidazole-urea derivatives with phenyl substitution **3a** (IC_50_ = 40.7 ± 0.06 µM for α-amylase; IC_50_ = 42.97 ± 0.19 µM for α-glucosidase), naphthyl substitution **3b** (IC_50_ = 41.7 ± 0.06 µM for α-amylase; IC_50_ = 45.97 ± 0.19 µM for α-glucosidase), and di-cyclohexyl urea substitution **3h** (IC_50_ = 43.04 ± 0.02 µM for α-amylase inhibition; IC_50_ = 44.99 ± 0.09 µM for α-glucosidase) manifested weak activities for both α-amylase and α-glucosidase inhibition.

Amongst the other benzimidazole urea derivatives, compound **3d** bearing a cyclohexyl side on nitrogen (IC_50_ = 25.65 ± 0.03 µM α-amylase inhibition; IC_50_ = 27.47 ± 0.13 µM α-glucosidase inhibition) and compound **3f** containing a butyl chain on nitrogen (IC_50_ = 28.33 ± 0.02 µM α-amylase inhibition; IC_50_ = 29.01 ± 0.12 µM α-glucosidase inhibition) exerted moderate activities for both α-amylase and α- glucosidase inhibition.

Derivative **3c** having a methoxy phenyl group displayed a significant α-amylase and α-glucosidase inhibitory activity. Substrate **3e** with an isopropyl group and **3g** substituted with a di-phenyl urea group on nitrogen also exhibited good inhibitory potential compared to standard acarbose.

**Figure 4:**
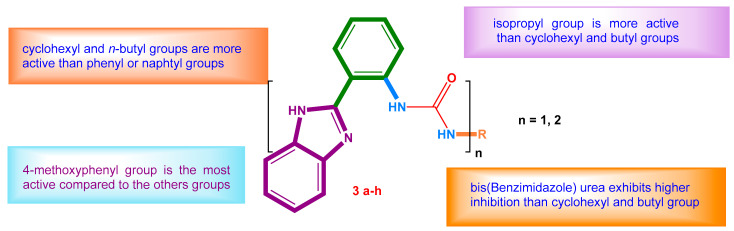
Structure-activity relationships related to benzimidazole urea derivatives **3a-h**.

In summary, in the series of aromatic substituents, compounds containing phenyl group **3a** and naphthyl group **3b** exhibited a weak activity for inhibition of both α-glucosidase and α-amylase. The excellent results were shown with target analog **3c** bearing 4-methoxyphenyl, showing remarkable inhibitory action against both α-amylase and α-glucosidase. The inclusion of a methoxy substituent in the phenyl group at the para-position significantly improved activity, which might be attributed to the favorable interaction for the inhibition activity of both α-amylase and α-glucosidase (Figure 4).

In the series of alkane and cycloalkane, it is clear that compounds **3d** and **3f** exhibited higher potential activity in comparison with aromatic compounds **3a** and **3b**. This observation is explained based on the donor effect of cyclohexyl and butyl groups when compared to phenyl or naphthyl groups. The same result was observed with compound **3e** bearing an isopropyl group manifesting a good inhibition of enzyme α-amylase and α-glucosidase. The above observation was interpreted by the sufficient donor effect of the isopropyl group, which exerted a favorable interaction and demonstrated the highest activity. Compound **3g** also indicated a strong inhibition of α-amylase and α-glucosidase compared with **3d** and **3f**. The presence of two phenylbenzimidazoles in the structure of compound **3g** allowed favorable interactions involving a good inhibition activity of α-amylase and α-glucosidase (Figure 4).

### 2.3. In Silico Docking Study

**Figure 5:**
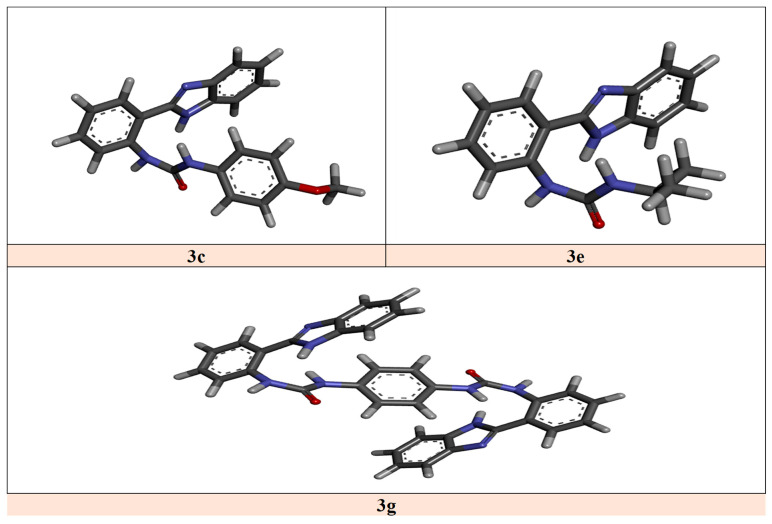
Optimized structures of **3c**, **3e**, and **3g** with Ammp calculations.

The objective of the in-silico study was to clarify and rationalize the type of interactions of the new benzimidazole urea **3a-h** in the target catalytic site. Molecular docking is a useful tool in the design of structure-based drugs. The optimized structures of the most potent benzimidazole urea **3c**, **3e**, and **3g** used in the docking study are presented in Figure 5. We notice that the most stable structures for **3c** and **3e** had a cis structure with intramolecular interactions. The most stable structure of **3g** had almost a Ci symmetry also with intramolecular interactions.

Benzimidazole urea derivatives showed good values of binding energies with α-amylase (HPA) human pancreatic and also the human lysosomal acid-α-glucosidase (HLAG) (Table 4).

**Figure 6:**
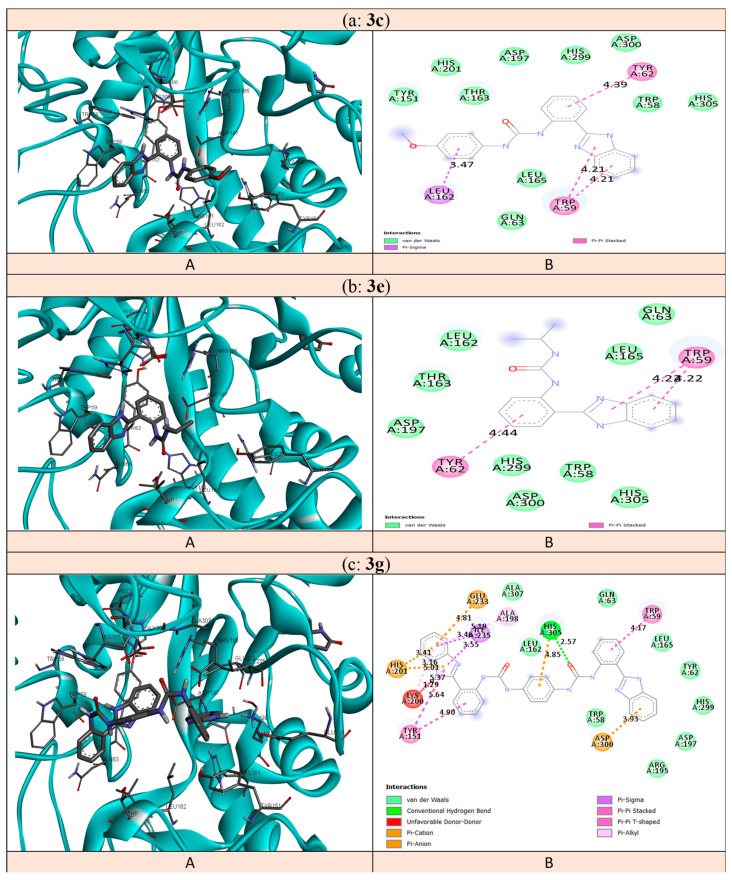
The interaction types of (**a**) **3c**, (**b**) **3e**, and (**c**) **3g** with surrounding amino acids of HPA are shown in 3D (**A**) and 2D (**B**).

**Figure 7:**
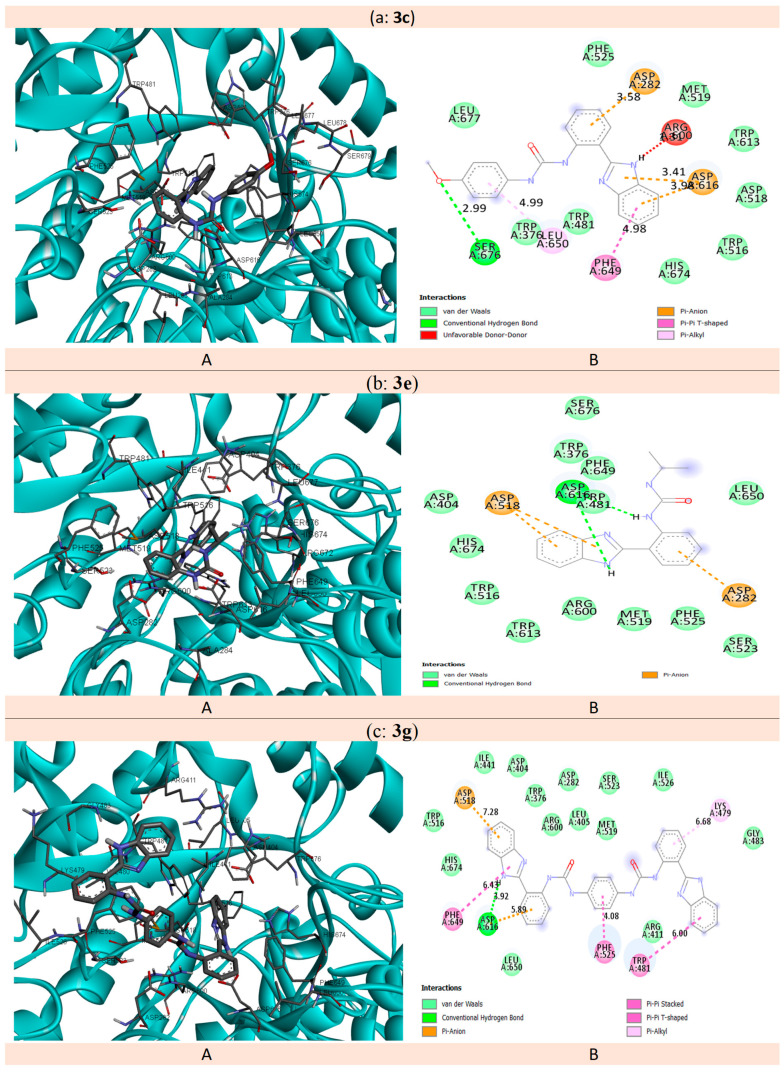
The interaction types of (**a**) **3c**, (**b**) **3e**, and (**c**) **3g** with surrounding amino acids of HLAG are shown in 3D (**A**) and 2D (**B**).

Our study was interested in explaining the interactions of the most effective analogs molecules **3c**, **3e**, and **3g** manifesting appropriate interactions (2D) and (3D) with the different amino acids illustrated in Figure 6 and Figure 7. Due to their high inhibition activity amongst molecules, the structures **3c**, **3e**, and **3g** were chosen for docking studies with 5E0F. Table 4 reveals that compounds **3c**, **3e**, and **3g** with potent inhibitory activities occupied suitable positions at the binding center of HPA, with energies of −8.8, −8.0, and −11.2 kcal.mol^−1^, respectively.

Presented in Figure 6, the structure of **3c** presented (π-σ) interaction with Leu162 (3.47 Å), two (π-π) interactions with Trp59, and one (π-π) interaction with Tyr62 (4.39 Å). The same type of interactions: two (π-π) interactions with Trp59 and one (π-π) interaction with Tyr62 (4.44 Å), existed in molecule **3e**. Compound **3g** presented many interactions with the protein: one hydrogen bond with His305 (2.57 Å); (π-ion) interactions with Glu233, His201, and Asp300; (π-π) interactions with Tyr151 and Trp59; (π-σ) interaction with Ile235; (π-Alkyl) interaction with Ala235.

The study of Ramasubbu et al. [73] showed that residues Asp 236, Glu233, Arg 61, Asp300, Lys 200, Leu165, and Asp197, and some of the aromatic and non-polar residues, Tyr 258, Ile235, His305, His299, Trp58, Ala307, Tyr62, Trp59, Ser163, and His101, defined the catalytic site of α-amylase. Williams and coworkers [74] reported that the inhibition property of myricetin with α-amylase has an appropriate position in the catalytic site of α-amylase through four hydrogen bonds with Gln63, His101, and Asp197, and possessing two hydrophobic interactions with Leu165 and Tyr62.

The data indicated that the structures of **3c**, **3e**, and **3g** had interactions with the catalytic site through Trp59, Trp58, Leu165, Tyr62, and Gln63 remaining from the catalytic HPA site. This confirms that **3c**, **3e**, and **3g** are good inhibitors for HPA.

The compounds **3c**, **3e**, and **3g** showing high inhibitory effects with HLAG gave important values of binding energies with the catalytic site of 5NN8, as mentioned in Table 4. These values were −8.2, −7.6, and −10.0 kcal mol^−1^, respectively.

Illustrated in Figure 7, the structure 3c had one hydrogen bond with Ser676 (2.99 Å); (π-anion) interactions with Asp282 and Asp616; (π-π) interaction with Phe649; (π-Alkyl) interaction with Leu650. The molecule **3e** had two hydrogen bonds with Asp616 (2.64 Å and 2.69 Å) and (π-anion) interactions with Asp518 and Asp282. Compound **3g** had one hydrogen bond with Asp616; (π-anion) interaction with Asp518; three (π-π) interactions with Phe649, Phe525, and Trp481.

Roig-Zamboni et al. [75] observed that when 1-deoxynojirimycin is inserted into the catalytic site of HLAG, it has a high inhibitory activity. This molecule has interactions with Trp376, Asp404, Leu405, Ile441, Trp481, Trp516, Asp518, Met519, Arg600, Trp613, Asp616, Asp645, Arg672, His674, and Phe649. Our data indicate that our structures **3c**, **3e**, and **3g** interacted with the HLAG catalytic site via many similar residues (Trp376, His674, Trp481, Trp376, Trp516, Trp481, Trp516, Asp518, Met519, Asp518, Arg600, Asp518, and Asp616). The different complexes of **3c**, **3e**, and **3g** were stabilized by these interactions and suggests that this stabilization contributed to the inhibitory activity.

## 3. Conclusions

A prominent series of benzimidazole-urea analogs **3a-h** was designed and synthesized from 2-(1*H*-benzoimidazol-2-yl) aniline **1** and the appropriate isocyanates **2a-h**. The antioxidant and antidiabetic activities of the target benzimidazole-ureas **3a-h** were evaluated. When measured using four different methods, the antioxidant data revealed that the most desired benzimidazole-urea exhibited high activity. The results indicated that among the series, compounds **3a** and **3b** displayed good inhibition with three methods: FRAP, MCA, and DPPH-SA. The most potent antioxidant compound was **3g**, which demonstrated strong activity in all four methods. Furthermore, the ability of benzimidazole-urea scaffolds to inhibit α-glycosidase and α-amylase was investigated. According to our predictions, the majority of tested compounds exhibited good to moderate activities. The results indicated that compounds **3e** and **3g** exhibited good inhibition for both enzymes, α-amylase, and α-glucosidase. Compound **3c** bearing the 4-methoxy moiety on the phenyl group at the para-position was found to be a more potent inhibitor of α-amylase, and α-glucosidase similar to the standard drug acarbose. The study confirms that compounds **3c**, **3e**, and **3g** have strong in vitro antioxidant properties, as well as α-glucosidase and α-amylase inhibition activities comparable to those of acarbose, suggesting that they could be used as anti-diabetic medications.

## 4. Experimental

### 4.1. General Details

Without any further purification, all commercially available reagents were used. Unless otherwise specified, all reactions were carried out in a dry nitrogen-protected atmosphere using oven-dried glassware. FT-IR spectra were recorded on an Agilent (Agilent Technologies, Inc. Headquarters, address: 5301 Stevens Creek Blvdv, Santa Clara, CA 95051, USA) apparatus type Cary 600 in the range of 400–4000 cm^−1^. A Bruker (Bruker Daltonics Inc., Bremen, Germany) NMR spectrometer Avance III HD 400 MHz was used to analyze ^1^H and ^13^C NMR spectra at 400 and 100 MHz, respectively. All spectra were recorded and referenced to TMS using DMSO *d_6_* as the solvent. According to an internal standard of residual DMSO *d_6_* at 2.50 ppm, chemical shifts in ^1^H NMR spectra were mentioned in parts per million (ppm) on the scale. Spin multiplicities are denoted by the letters s (singlet), brs (broad singlet), d (doublet), t (triplet), q (quartet), and m (multiplet). Chemical shifts in ^13^C NMR spectra were also indicated in ppm from the peak position of DMSO *d_6_* (39.52 ppm). The values of the coupling constant *J* are given in hertz (Hz). High-resolution mass spectra were recorded on an Agilent 6545 LC/Q-TOF MS (Agilent Technologies, Inc. Headquarters, address: 5301 Stevens Creek Blvd, Santa Clara, CA 95051, USA). Using the Stuart SMP30 apparatus (New Jersey 07740, États-Unis), the melting points in open capillary tubes were determined. For analytical TLC, Silica Gel 60 F254 plates (Sigma 40–60 m) were employed, and the chromatogram was developed using a UV lamp 254 nm. DMSO-*d_6_* (99.9 atom % D; CAS no. 2206-27-1) were purchased from Sigma-Aldrich and used without further purification.

### 4.2. Chemistry: Benzimidazole-Ureas ***3a–h*** Synthesis: General Procedure

The reaction was carried out under a nitrogen atmosphere. In a 100 mL round bottom flask, 0.52 g (2.5 mmol) of 2-(1*H*-benzoimidazol-2-yl) aniline was placed in 20 mL of dichloromethane and 5 mL of acetone, and the appropriate isocyanate (3.75 mmol) was added dropwise in 20 mL of dichloromethane and 5 mL of acetone. The reaction was stirred under reflux. TLC and diethyl ether/hexane (50/50) as the eluent were used to monitor the reaction progress (see Table 3). After filtering the reaction mixture, the raw solid was purified through recrystallization from ethanol (see Supplementary Materials).

1-(2-(1*H*-benzo[d]imidazol-2-yl)phenyl)-3-phenylurea **3a**: White precipitate*,* yield: 84%; M.p. = 129–131 °C; IR (cm^−1^) *ν*: 3249 (NH), 1672 (C=O), 1586 (C=N), 1536 (C=C); ^1^H NMR (400 MHz, DMSO-*d_6_*) *δ*(ppm): 13.08 (s, 1H, NH), 12.02 (s, 1H, NH), 9.69 (s, 1H, NH), 8.40 (d, 1H, *J* = 8.44 Hz, H_arom_), 7.78 (m, 1H, H_arom_), 7.56 (d, 3H, *J* = 7.68 Hz, H_arom_), 7.45 (td, 1H, *J* = 8.44 Hz, H_arom_), 7.33–7.28 (m, 1H, H_arom_), 7.17 (td, 1H, *J* = 8.00 Hz, H_arom_); 7.01 (t, 1H, *J* = 7.36 Hz, H_arom_); ^13^C NMR (75 MHz, DMSO-*d_6_*) *δ*(ppm): 153.2 (C=O), 151.45 (C=N), 142.7, 140,3, 139.8, 134.1, 130,7, 129.1, 127.7, 123.6, 122.6, 122.4, 121.7, 120.9, 119.7, 119.3, 115.7, 111.8; HRMS: *m*/*z* for C_20_H_16_N_4_O calcd: 328.1324, found: 328.12280 [M]^+^.

1-(2-(1*H*-benzo[d]imidazol-2-yl)phenyl)-3-(naphthalen-1-yl)urea **3b**: White precipitate*,* yield: 90%; M.p. = 203–205 °C; IR (cm^−1^) *ν*: 3394 (NH), 1647 (C=O), 1586 (C=N), 1518 (C=C); ^1^H NMR (400 MHz, DMSO-*d_6_*) *δ*(ppm): 13.01 (s, 1H, NH), 12.04 (s, 1H, NH), 9.45 (s, 1H, H_arom_), 8.50 (d, 1H, *J*= 8.48 Hz, H_arom_), 7.72–7.71 (dd, 1H, H_arom_), 8.03–7.95 (m, 2H, H_arom_), 7.85 (d, 1H, H_arom_), 7.56 (d, 1H, *J* = 7.44 Hz, H_arom_); 7.53–7.51 (m, 4H, H_arom_), 7.53 (td, 2H, *J* = 8.48 Hz, H_arom_), 7.22–7.13 (m, 3H, H_arom_); ^13^C NMR (75 MHz, DMSO-*d_6_*) *δ*(ppm): 154.6 (1C, C=O), 151.3 (C=N), 139.9, 134.6, 134.4, 130.7, 129.6, 128.5, 127.8, 126.5, 126.36, 126.34, 125.9, 123.5, 123.4, 122.22, 121.7, 120.7, 115.75, 111.6; HRMS: *m*/*z* for C_24_H_18_N_4_O calcd: 378.1481, found: 378.33484 [M]^+^.

1-(2-(1*H*-benzo[d]imidazol-2-yl)phenyl)-3-(4-methoxyphenyl)urea **3c**: White precipitate*,* yield: 80%; M.p. = 197–199 °C; IR (cm^−1^) *ν*: 3405 (NH), 1646 (C=O), 1587 (C=N), 1533 (C=C); ^1^H NMR (400 MHz, DMSO-*d_6_*) *δ*(ppm): 13.11 (s, 1H, NH), 12.08 (s, 1H, NH), 9.49 (s, 1H, NH), 8.53 (d, 1H, *J* = 8.3 Hz, H_arom_), 8.11 (d, 1H, *J* = 7.16 Hz, H_arom_), 7.80–7.78 (m, 1H, H_arom_), 7.63–7.61 (m, 1H, H_arom_), 7.54–7.45 (m, 3H, H_arom_), 7.41–7.31 (m, 2H, H_arom_), 7.17 (t, 1H, *J* = 7.28 Hz, H_arom_), 6.95 (d, 1H, *J* = 8.96 Hz, H_arom_); 6.90 (d, 1H, *J* = 9.00 Hz, H_arom_), 3.76 (s, 3H, OCH_3_); ^13^C NMR (75 MHz, DMSO-*d_6_*) *δ*(ppm): 154.8 (C=O), 153.6 (C=N), 153.5, 151.5, 142.8, 140.1, 134.1, 130.7, 127.7, 123.6, 122.3, 121.5, 120.7, 119.3, 115.8, 115.2, 114.9, 114.7, 114.0, 111.8, 55.5; HRMS: *m*/*z* for C_21_H_18_N_4_O_2_ calcd: 358.1430, found: 358.33315 [M]^+^.

1-(2-(1*H*-benzo[d]imidazol-2-yl)phenyl)-3-cyclohexylurea **3d**: White precipitate, yield: 75%; M.p. = 330–332 °C; IR (cm^−1^) *ν*: 3335 (NH), 1654 (C=O), 1587 (C=N), 1531 (C=C); ^1^H NMR (400 MHz, DMSO-*d_6_*) *δ*(ppm): 13.04 (s, 1H, NH), 11.67 (s, 1H, NH), 8.52 (d, 1H, *J* = 8.36 Hz, H_arom_), 8.02 (d, 1H, *J* = 7.36 Hz, H_arom_), 7.80–7.78 (m, 1H, H_arom_), 7.59–7.57 (m, 1H, H_arom_), 7.39 (t, 1H, *J* = 7.40 Hz, H_arom_); 7.28–7.27 (m, 2H, H_arom_), 7.08 (t, 2H, *J* = 7.24 Hz, H_arom_), 3.58 (m, 1H, CHN), 1.91–1.89 (m, 2H, CH_2_), 1.75–1.60 (m, 3H), 1.37–1.22 (m, 5H); ^13^C NMR (75 MHz, DMSO-*d_6_*) *δ*(ppm): 154.9 (C=O), 151.7 (C=N), 140.7, 134.1, 130.6, 127.7, 123.5, 122.3, 120.8, 128.0, 119.1, 116.5, 115.4, 114.8, 111.7; 55.3, 49.2, 33.5, 25.7, 25.4; HRMS: *m*/*z* for C_20_H_22_N_4_O calcd: 334.1794, found: 334.19339 [M]^+^.

1-(2-(1*H*-benzo[d]imidazol-2-yl)phenyl)-3-isopropylurea **3e**: White precipitate, yield: 65%; M.p. = 196–198 °C; IR (cm^−1^) *ν*: 3355 (NH), 1654 (C=O), 1587 (C=N), 1532 (C=C); ^1^H NMR (400 MHz, DMSO-*d_6_*) *δ*(ppm): 13.07 (s, 1H, NH), 11.73 (s, 1H, NH), 8.53 (d, 1H, *J* = 8.40 Hz, H_arom_), 8.03 (d, 1H, *J* = 7.32 Hz, H_arom_), 7.78 (m, 1H, H_arom_), 7.58 (m, 1H, H_arom_), 7.39 (t, 1H, *J* = 7.40 Hz, H_arom_); 7.28–7.27 (m, 2H, H_arom_), 7.08 (t, 2H, *J* = 7.28 Hz, H_arom_), 3.95–3.90 (m, 1H, CHN), 1.19 (s, 6H, 2CH_3_); ^13^C NMR (75 MHz, DMSO-*d_6_*) *δ*(ppm): 154.9 (C=O), 153.0 (C=N), 151.7, 148.7, 142.7, 140.7, 134.0, 130.6, 127.7, 123.5, 122.7, 122.3, 121.8, 120.8, 119.9, 119.2, 118.6, 116.5, 115.4, 114.7, 111.7, 111,2, 110.5; HRMS: *m*/*z* for C_17_H_30_O_2_N_2_ calcd: 294.23073, found: 294.23016 [M]^+^.

1-(2-(1H-benzo[d]imidazol-2-yl)phenyl)-3-butylurea **3f**: White precipitate, yield: 60%; M.p. = 196–198 °C; IR (cm^−1^) *ν*: 3407 (NH), 1651 (C=O), 1510 (C=N), 1512(C=C); ^1^H NMR (400 MHz, DMSO-*d_6_*) *δ*(ppm): 7.91 (dd, 1H, *J* = 7.64 Hz, H_arom_), 7.74–7.71 (m, 1H, H_arom_), 7.65–7.63 (m, 1H, H_arom_), 6.94 (m, 2H, NH_2_), 6.82–6.78 (m, 2H, H_arom_), 3.61 (m, 2H, CH_2_N), 1.19 (s, 6H, 3CH_2_); ^13^C NMR (75 MHz, DMSO-*d_6_*) *δ*(ppm): 148.7, 144.6, 143.4.0, 132.7, 132.0, 125.1, 122.5, 122.3, 121.8, 119.3, 118.4, 116.5, 115.4, 112.3, 111,9, 110.5, 72.3, 28.4; HRMS: *m*/*z* for C_18_H_20_N_4_O calcd: 308.1637, found: 308.18590 [M]^+^.

1,1’-(1,4-phenylene)bis(3-(2-(1H-benzo[d]imidazol-2-yl)phenyl)urea) **3g**: White precipitate, yield: 93%; M.p. = 190–192 °C, IR (cm^−1^) *ν*: 3326 (NH), 1652 (C=O), 1588 (C=N), 1527 (C=C); ^1^H NMR (400 MHz, DMSO-*d_6_*) *δ*(ppm): 13.05 (s, 2H, 2NH), 11.97 (s, 2H, 2NH), 9.53 (s, 2H, NH), 8.45 (d, 2H, *J* = 6.35 Hz, H_arom_), 8.05 (d, 2H, *J* = 6.35 Hz, H_arom_), 7.78–7.69 (m, 2H, H_arom_), 7.58–7.57 (m, 2H, H_arom_), 7.50–7.41 (m, 12H, H_arom_); ^13^C NMR (75 MHz, DMSO-*d_6_*) *δ*(ppm): 153.45, (C=O), 151.47 (C=N), 142.7, 139.9, 142.7, 140.7, 134.9, 134.1, 130.7, 127.7, 123.5, 122.3, 121.61, 120.8, 120.7, 119.4, 119.1, 115.6, 114.5; HRMS: *m*/*z* for C_34_H_26_N_8_O_2_ calcd: 578.2179, found: 578.20871 [M]^+^.

1,1′-(cyclohexane-1,4-diylbis(methylene))bis(3-(2-(1H-benzo[d]imidazol-2-yl) phenyl) urea) **3h**: White precipitate, yield: 85%; M.p. = 190–192 °C, IR (cm^−1^) *ν*: 3410 (NH), 1651 (C=O), 1587 (C=N), 1512 (C=C); ^1^H NMR (400 MHz, DMSO-*d_6_*) *δ*(ppm): 13.01 (s, 2H, 2NH), 11.8–11.5 (s, 2H, 2NH), 8.58–8.45 (m, 2H, H_arom_), 8.07–7.88 (m, 2H, H_arom_), 7.85–7.33 (m, 8H, H_arom_), 7.32–7.14 (m, 6H, H_arom_), 7.14–7.87 (m, 4H, H_arom_), 3.1–3.9 (m, 4H, 2CH_2_N), 0.5–2.1 (m, 10H); ^13^C NMR (75 MHz, DMSO-*d_6_*) *δ*(ppm): 155.1, 155.0 (C=O), 151.7, 151.6 (C=N), 142.8, 140.6, 140.5, 134.1, 130.8, 130.6, 127.8, 127.7, 123.4, 122.4, 120.9, 120.8, 120.3, 120.0, 119.1, 116.5, 115.4, 115.1, 114.8, 111,8, 49.7. 48.8; 47.2, 44.6, 34.0, 33.9, 33.3, 32.5, 31.7, 29.5, 28.4, 28,2; HRMS: *m*/*z* for C_36_H_36_N_8_O_2_ calcd: 612.2961, found: 612.34533 [M]^+^.

### 4.3. Biology

#### 4.3.1. Antioxidant Study

##### Total Antioxidant Capacity (TAC)

An amount of 2 mL of freshly prepared 0.6 M solution of sulfuric acid, a solution of ammonium molybdate (4 mM), and a buffer solution of sodium phosphate (28 mM) were placed in small glass test tubes and thoroughly mixed with the compound solution (200 μL in DMSO with a concentration of 1 mM) [76]. An amount of 2 mL of molybdate reagent was mixed with 200 μL of distilled water to make a blank test. The cylinders were warmed to 85 °C for 1.5 h before cooling to ambient temperature. At 695 nm, the produced blue color was assessed with a UV spectrophotometer, with the blank reading having to serve as the auto-zero point. The TAC results were calculated using the Trolox standard calibration curve.

##### DPPH Scavenging Activity (DPPH-SA)

The ability of the compounds to scavenge free radicals was tested against DPPH-stable free radicals. The degree of reduction in the DPPH violet color is directly proportional to the scavenging efficacy of the compounds. According to the method of Aroua et al. [52], 1 mL of the DMSO solutions of the tested compounds (1 mM final concentration) was thoroughly mixed in test tubes with the DPPH solution prepared at the concentration of 300 µM in methanol. After 30 min at room temperature, the reduction in DPPH-violet color was measured at 517 nm. The DPPH-SA of the compounds was calculated using the DPPH-Trolox calibration curve.

##### Ferric Reducing Antioxidant Power (FRAP) Assay

The FRAP reagent is a 1:1:10 mixture of TPTZ (2,4,6-Tris(2-pyridyl)-S-triazine, 10 mM prepared in 40 mM HCl), FeCl_3_.6H_2_O (20 mM), and acetate buffer (300 mM, pH 3.6) [77]. Accurately, 0.1 mL of the samples (final concentration 1 mM in DMSO) was thoroughly mixed with 2 mL of the FRAP reagent and incubated at room temperature for 30 min. At 593 nm, the absorbance was measured. The compounds FRAP activity was calculated using the FRAP-trolox calibration curve in trolox equivalent.

##### Iron Chelating Activity Assay (MCA)

Using the method of Zengin et al. [78] with modification, the ability of compounds to chelate iron compared to the EDTA was estimated. To inchoate the color, a compound solution in DMSO (2 with a final concentration of 1 mM) plus ferrous chloride (25 μL, 2 mM, FeCl_2_) was added to 100 μL of ferrozine. At 562 nm, the absorbance of the mixture was measured in comparison to a blank (prepared similarly to a test without ferrozine). The chelating activity of the extract was calculated in equivalents to the EDTA standard calibration curve.

#### 4.3.2. α-Amylase and α-Glucosidase Inhibition Assay

##### α-Amylase Inhibition Assay

The α-amylase inhibition activity was measured using the previously reported methodologies [52]. An amount of 500 μL of test sample (10–1000 M) and 500 μL of substrate α-amylase (source: *Aspergillus oryzae*) solution (0.5 mg/mL) in 0.2 mM phosphate buffer (pH 6.9) were incubated for 10 min at 25 °C. Following pre-incubation, 500 μL of 1% starch solution in 0.02 M sodium phosphate buffer (pH 6.9) was added and incubated for 10 min at 25 °C. An amount of 1 mL of dinitro salicylic acid color reagent was used to stop the reaction. The tubes were then placed in boiling water for 5 min before being cooled to room temperature. After diluting the solutions with 10 mL of distilled water, the absorbance at 540 nm was measured. The standard medication was acarbose [79]. The percentage of enzyme inhibition was calculated using the following equation:

(1) $$\%\;\mathrm{of}\;\mathrm{Enzyme}\;\mathrm{Inhibition}=\frac{\left(\mathrm{Absorbance}\;\mathrm{of}\;\mathrm{control}\;–\;\mathrm{Absorbance}\;\mathrm{of}\;\mathrm{compound}\right)\times 100\;}{\mathrm{Absorbance}\;\mathrm{of}\;\mathrm{control}}$$

The IC_50_ values, concentrations necessary to reduce α-amylase activity by 50%, were measured and used Graph Pad Prism Software and a non-linear correlation graph plotting percentage inhibition (x axis) versus concentrations (y axis) (Version 5).

##### α-Glucosidase Inhibition Assay

The inhibition of α-glucosidase activity was measured using a modified version of a previously published method [80]. In 100 mL of phosphate buffer (pH 6.8) containing 200 mg of bovine serum albumin, one milligram of α-glucosidase (Saccharomyces cerevisiae, Sigma-Aldrich, St. Louis, MO, USA) was dissolved (Merck, Darmstadt, Germany). The reaction mixture was made up of 10 μL of sample at various concentrations (10–1000 M), 490 μL of phosphate buffer pH 6.8, and 250 μL of 5 mM *p*-nitrophenyl-D-glucopyranoside (Sigma-Aldrich, Buchs, Switzerland). After a 5 min preincubation at 37 °C, 250 μL of α-glucosidase (0.15 unit/mL) was added and incubated at 37 °C for 15 min. The reaction was stopped by adding 2000 μL Na_2_CO_3_ 200 mM. α-glucosidase activity was measured using a spectrophotometer UV-Vis (Shimadzu 265, Kyoto, Japan) at 400 nm by measuring the amount of *p*-nitrophenol released from *p*-NPG. As a positive control for α-glucosidase inhibitor, acarbose was used [81]. The IC_50_ value was defined as the concentration of the extract required to inhibit 50% of α-glucosidase activity under the assay conditions. Equation (1) was used to calculate the percentage of inhibition.

### 4.4. Statistical Analysis

The IC_50_ values, concentrations necessary to inhibit the α-amylase and α-glucosidase activities by 50%, were determined using Graph Pad Prism Software (Version 5) and a non-linear regression graph plotting percentage inhibition (x axis) versus concentrations (y axis).

### 4.5. Molecular Docking

The new benzimidazole-urea **3a-h** and their interactions with HPA and HLAG were studied by molecular docking in order to clarify the appropriate position for the ligands in the catalytic site of the proteins. We imported HPA (PDB code: 5E0F) and HLAG (PDB code: 5NN8) from the Protein Data Bank. Molecules of water and co-crystallized ligand from the receptors were removed. AutoDockTools1.5.2 (ADT) was used to add Gasteiger charges and polar hydrogens to the files, which were then converted to PDBQT format [82,83]. A docking grid was chosen by ADT. The grid box in HPA was centered at x = −7.946 Å, y = 10.438 Å, and z = −21.863 Å using a grid of x = 80, y = 72, and z = 66 points. The grid box for HLAG was centered at x = −12.175 Å, y = −35.415 Å, and z = 88.753 Å using a grid of x = 74, y = 70, and z = 90 points. A spacing of 0.375 Å was chosen.

The optimization of the compounds was performed using a conjugate gradient AMMP implemented in VEGA ZZ software [84]. The file format was converted from PDB to PDBQT using ADT. The docking calculations were performed by AutoDock Vina software [85] with an exhaustiveness parameter of 32. ADT allowed the analysis of the conformations obtained from the docking calculations. The ligand–receptor interaction types were determined using Discovery Studio Visualizer [86].

## Figures and Tables

**Table 1:** Benzimidazole-ureas **3a-h** synthesis from 2-(1*H*-benzoimidazol-2-yl)aniline **1** and isocyanates **2a-h** ^a^.

Entry	Isocyanate 2	Benzimidazole Urea 3	Time ^b^ (h)	Yield ^c^ (%)
1	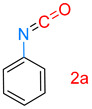	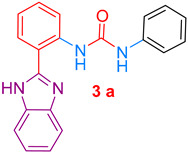	1	85
2	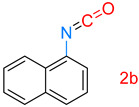	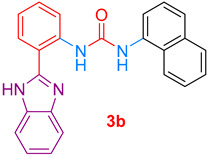	1	90
3	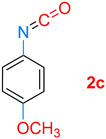	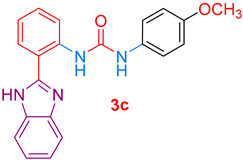	2	80
4	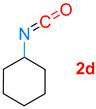	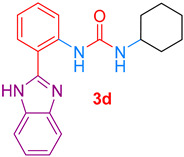	8	75
5	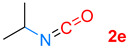	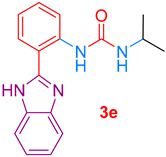	4	65
6	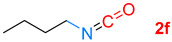	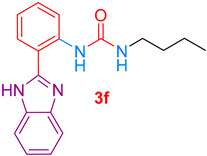	10	60
7	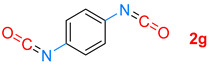	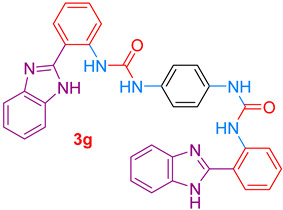	2	93
8	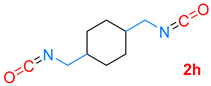	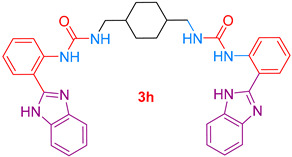	10	85

^a^ Reaction conditions: **1** (1 mmol, 1 equiv.); isocyanate **2** (1.5 mmol, 1.5 equiv.) in 40 mL of dichloromethane and 10 mL of acetone. ^b^ TLC (eluent: Ether/Hexane: 50/50) is used to determine reaction time. ^c^ Yields were measured after purification.

**Table 2:** Antioxidant activities of target benzimidazole-urea **3a-h**.

Compounds 3	TAC	DPPH-SA	FRAP	MCA
**3a**	1.33 ± 0.46	1.17 ± 0.07	6.53 ± 0.03	1.62 ± 0.10
**3b**	5.16 ± 0.69	0.96 ± 0.06	9.25 ± 0.20	1.55 ± 0.07
**3c**	4.43 ± 0.53	1.61 ± 0.02	8.65 ± 0.29	1.55 ± 0.22
**3d**	2.47 ± 0.42	2.89 ± 1.07	5.67 ± 0.69	1.46 ± 0.10
**3e**	2.52 ± 0.80	1.70 ± 0.01	2.75 ± 0.15	1.47 ± 0.09
**3f**	8.50 ± 0.87	1.55 ± 0.03	5.99 ± 0.02	1.40 ± 0.07
**3g**	10.06 ± 1.51	3.86 ± 0.04	16.12 ± 0.29	1.36 ± 0.09
**3h**	2.25 ± 0.65	1.09 ± 0.03	6.79 ± 0.50	2.95 ± 0.21

TAC: total antioxidant capacity (trolox equivalent per 1 mM of the compounds), DPPH-SA: 2,2-diphenyl-1-picrylhydrazyl scavenging activity (trolox equivalent per 1 mM of the compounds), FRAP: ferric reducing antioxidant power (trolox equivalent per 1 mM of the compounds), MCA: metal chelating activity (EDTA equivalent per 1 mM of the compounds).

**Table 3:** α-amylase and α-glucosidase inhibition activity of benzimidazole-urea **3a-h**.

Compounds 3	α-Amylase Inhibition IC_50_ ± SEM (µM*)*	α-Glucosidase Inhibition IC_50_ ± SEM (µM)
**3a**	40.7 ± 0.06	42.97 ± 0.19
**3b**	41.7 ± 0.06	45.97 ± 0.19
**3c**	18.65 ± 0.23	17.47 ± 0.03
**3d**	25.65 ± 0.03	27.47 ± 0.13
**3e**	20.7 ± 0.06	21.97 ± 0.19
**3f**	28.33 ± 0.02	29.01 ± 0.12
**3g**	22.33 ± 0.12	23.01 ± 0.12
**3h**	43.04 ± 0.02	44.99 ± 0.09
Acarbose	14.21 ± 0.02	15.41 ± 0.32

SEM = Standard error mean for triplicate values.

**Table 4:** Complexes with HPA and HLAG docking binding energies (kcal.mol^−1^).

Compounds 3	α-Amylase (E kcal/mol)	α-Glucosidase (E kcal/mol)
**3a**	−8.8	−8.7
**3b**	−10.3	−9.9
**3c**	−8.8	−8.2
**3d**	−8.6	−8.6
**3e**	−8.0	−7.6
**3f**	−7.8	−7.6
**3g**	−11.2	−10.0
**3h**	−10.4	−10.0

## Data Availability

All data are available in the manuscript and Supplementary File.

## Supplementary Materials

The following supporting information can be downloaded at: https://www.mdpi.com/article/10.3390/pharmaceutics15020457/s1, Figure S1: FTIR spectrum of compound **3a**; Figure S2: ^1^H-NMR spectrum of compound **3a**; Figure S3: ^13^C NMR spectrum of compound **3a**, Figure S4: HRMS spectrum of compound **3a**, Figure S5: FTIR spectrum of compound **3b**; Figure S6: ^1^H-NMR spectrum of compound **3b**; Figure S7: ^13^C NMR spectrum of compound **3b**, Figure S8: HRMS spectrum of compound **3b**, Figure S9: FTIR spectrum of compound **3c**; Figure S10: ^1^H-NMR spectrum of compound **3c**; Figure S11: ^13^C NMR spectrum of compound **3c**, Figure S12: HRMS spectrum of compound **3c**, Figure S13: FTIR spectrum of compound **3d**; Figure S14: ^1^H-NMR spectrum of compound **3d**; Figure S15: ^13^C NMR spectrum of compound **3d**, Figure S16: HRMS spectrum of compound **3d**, Figure S17: FTIR spectrum of compound **3e**; Figure S18: ^1^H-NMR spectrum of compound **3e**; Figure S19: ^13^C NMR spectrum of compound **3e**, Figure S20: HRMS spectrum of compound **3e**, Figure S21: FTIR spectrum of compound **3f**; Figure S22: ^1^H-NMR spectrum of compound **3f**; Figure S23: ^13^C NMR spectrum of compound **3f**, Figure S24: HRMS spectrum of compound **3f**, Figure S25: FTIR spectrum of compound **3g**; Figure S26: ^1^H-NMR spectrum of compound **3g**; Figure S27: ^13^C NMR spectrum of compound **3g**, Figure S28: HRMS spectrum of compound **3g**, Figure S29: FTIR spectrum of compound **3h**; Figure S30: ^1^H-NMR spectrum of compound **3h**; Figure S31: ^13^C NMR spectrum of compound **3h**, Figure S32: HRMS spectrum of compound **3h**.
