# Supplementary material for: Synthesis, Molecular Docking, and Bioactivity Study of Novel Hybrid Benzimidazole Urea Derivatives: A Promising α-Amylase and α-Glucosidase Inhibitor Candidate with Antioxidant Activity
Source: Pharmaceutics. 2023 Jan 30;15(2):457. doi: 10.3390/pharmaceutics15020457 (PMC9963656; doi:10.3390/pharmaceutics15020457)
Supplement: Supplementary file 1 [file pharmaceutics-15-00457-s001.zip › pharmaceutics-2138233-supplementary.pdf]

Supplementary Materials:

Synthesis, Molecular Docking, and Bioactivity Study of Novel Hybrid Benzimidazole Urea Derivatives: A Promising α-amylase and α-Glucosidase Inhibitor Candidate with Antioxidant Activity

Lotfi M. Aroua, Abdulelah H. Alosaimi, Fahad M Alminderej, Sabri Messaoudi, Hamdoon A. Mohammed, Suliman A. Almahmoud, Sridevi Chigurupati, Abuzar E. A. E. Albadri and Nejib H. Mekni

Table of Contents

**Figure S-1:** FTIR spectrum of compound **3a**	3
**Figure S-2**: ^{1}H-NMR spectrum of compound **3a**.	3
**Figure S- 3:** ^{13}C NMR spectrum of compound **3a**	4
**Figure S-4:** HRMS spectrum of compound **3a**	4
**Figure S-5:** FTIR spectrum of compound **3b**	5
**Figure S-6**: ^{1}H-NMR spectrum of compound **3b**.	5
**Figure S-7**: ^{13}C-NMR spectrum of compound **3b**.	6
**Figure S-8:** HRMS spectrum of compound **3b**	6
**Figure S-9:** FTIR spectrum of compound **3c**	7
**Figure S-10**: ^{1}H-NMR spectrum of compound **3c**.	7
**Figure S-11:** ^{13}C NMR spectrum of compound **3c**	8
**Figure S-12:** HRMS spectrum of compound **3c**	8
**Figure S-53:** FTIR spectrum of compound **3d**	9
**Figure S-14**: ^{1}H-NMR spectrum of compound **3d**.	9
**Figure S- 15:** ^{13}C NMR spectrum of compound **3d**	10
**Figure S-16:** HRMS spectrum of compound **3d**	10
**Figure S-67:** FTIR spectrum of compound **3e**	11
**Figure S-18**: ^{1}H-NMR spectrum of compound **3e**.	11
**Figure S-19:** ^{13}C NMR spectrum of compound **3e**	12
**Figure S-20:** HRMS spectrum of compound **3e**	12
**Figure S-27:** FTIR spectrum of compound **3f**	13
**Figure S-82**: ^{1}H-NMR spectrum of compound **3f**.	13
**Figure S-29:** ^{13}C NMR spectrum of compound **3f**	14
**Figure S-210:** HRMS spectrum of compound **3f**	14
**Figure S-25:** FTIR spectrum of compound **3g**	15
**Figure S-116**: ^{1}H-NMR spectrum of compound **3g**.	15
**Figure S-27:** ^{13}C NMR spectrum of compound **3g**	16
**Figure S-28:** HRMS spectrum of compound **3g**	16
**Figure S-29:** FTIR spectrum of compound **3h**	17
**Figure S-30**: ^{1}H-NMR spectrum of compound **3h**.	17
**Figure S- 121:** ^{13}C NMR spectrum of compound **3h**	18
**Figure S-32:** HRMS spectrum of compound **3h**	18

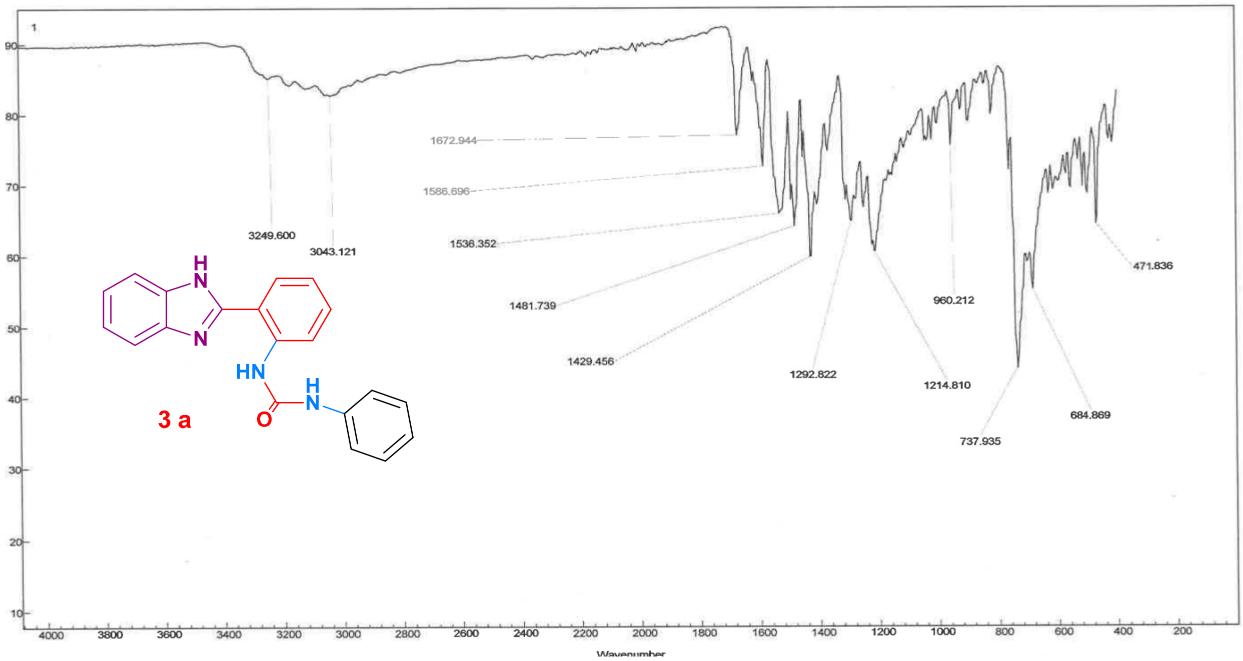

Figure S-1: FTIR spectrum of compound **3a**.

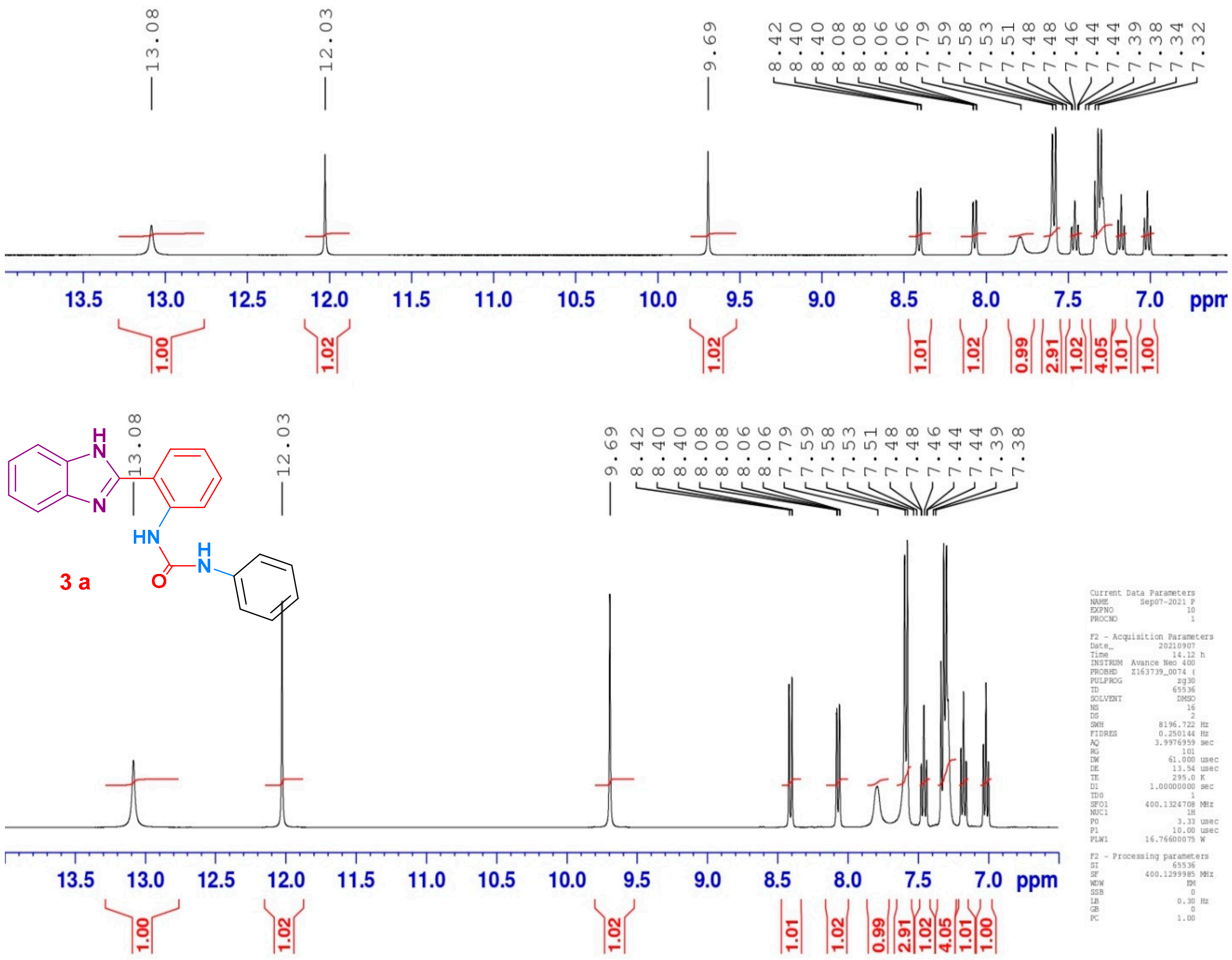

Figure S-2: ^{1}H-NMR spectrum of compound **3a**.

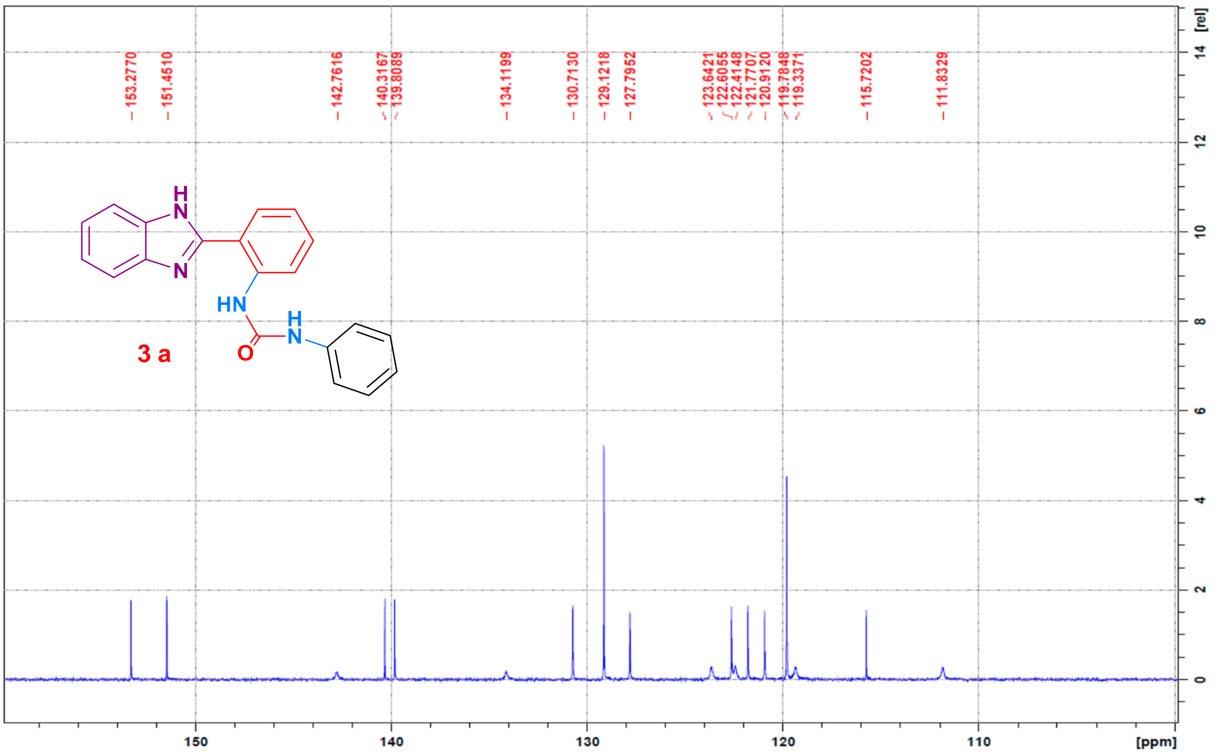

Figure S-3: ^{13}C NMR spectrum of compound **3a**.

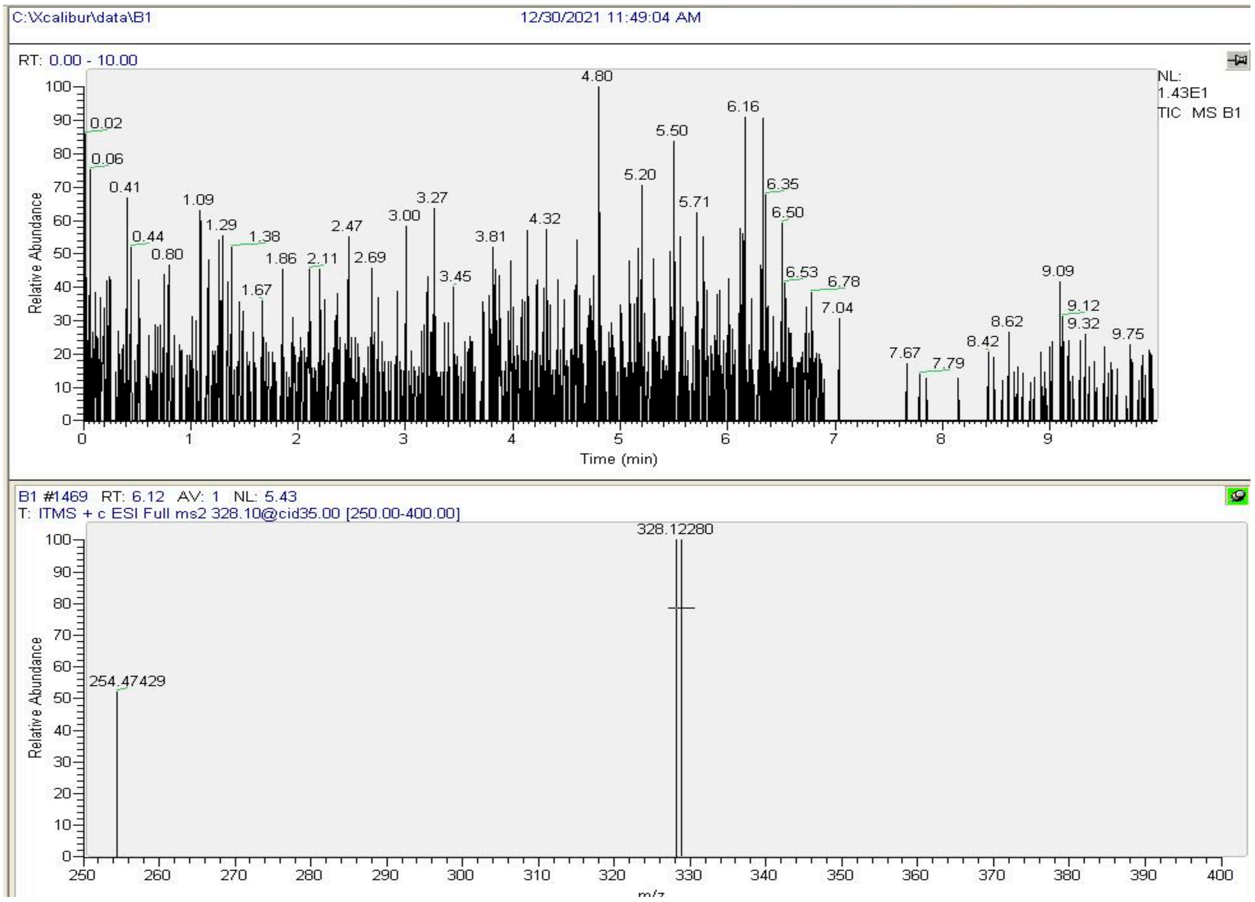

Figure S-4: Mass spectrum of compound **3a**.

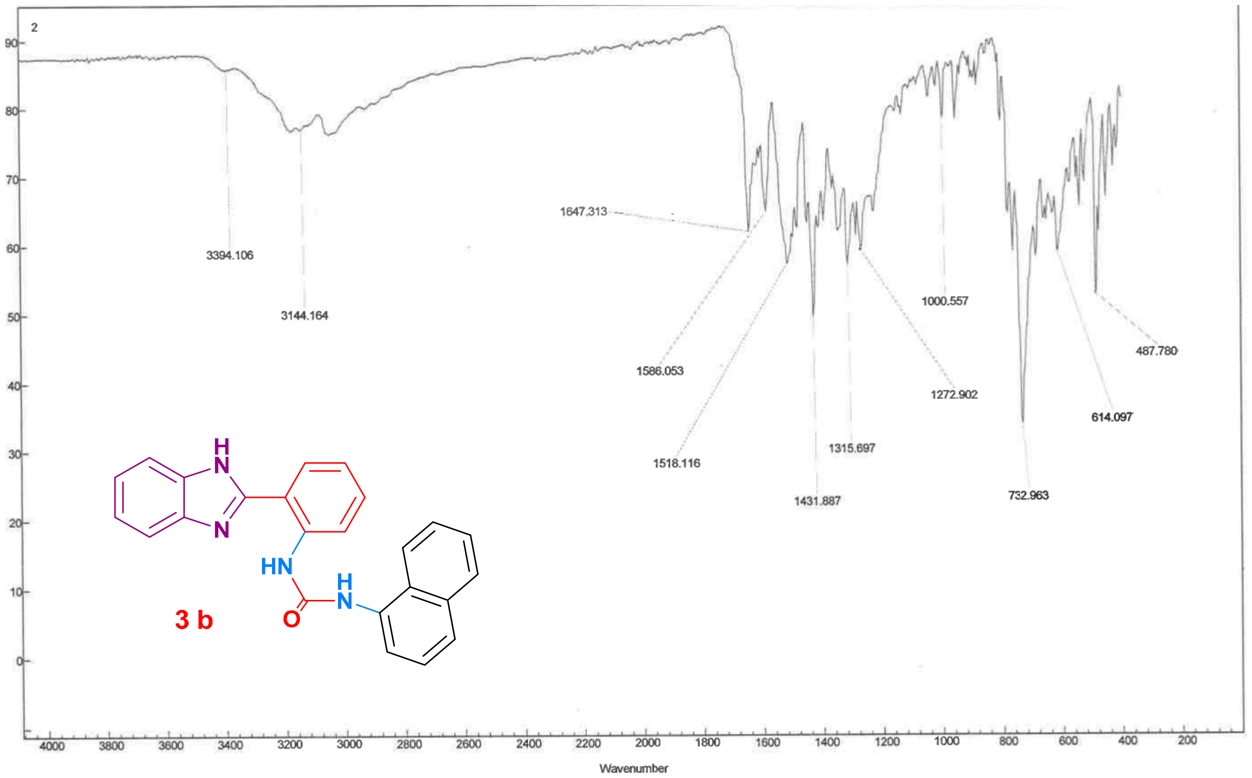

Figure S-5: FTIR spectrum of compound **3b**.

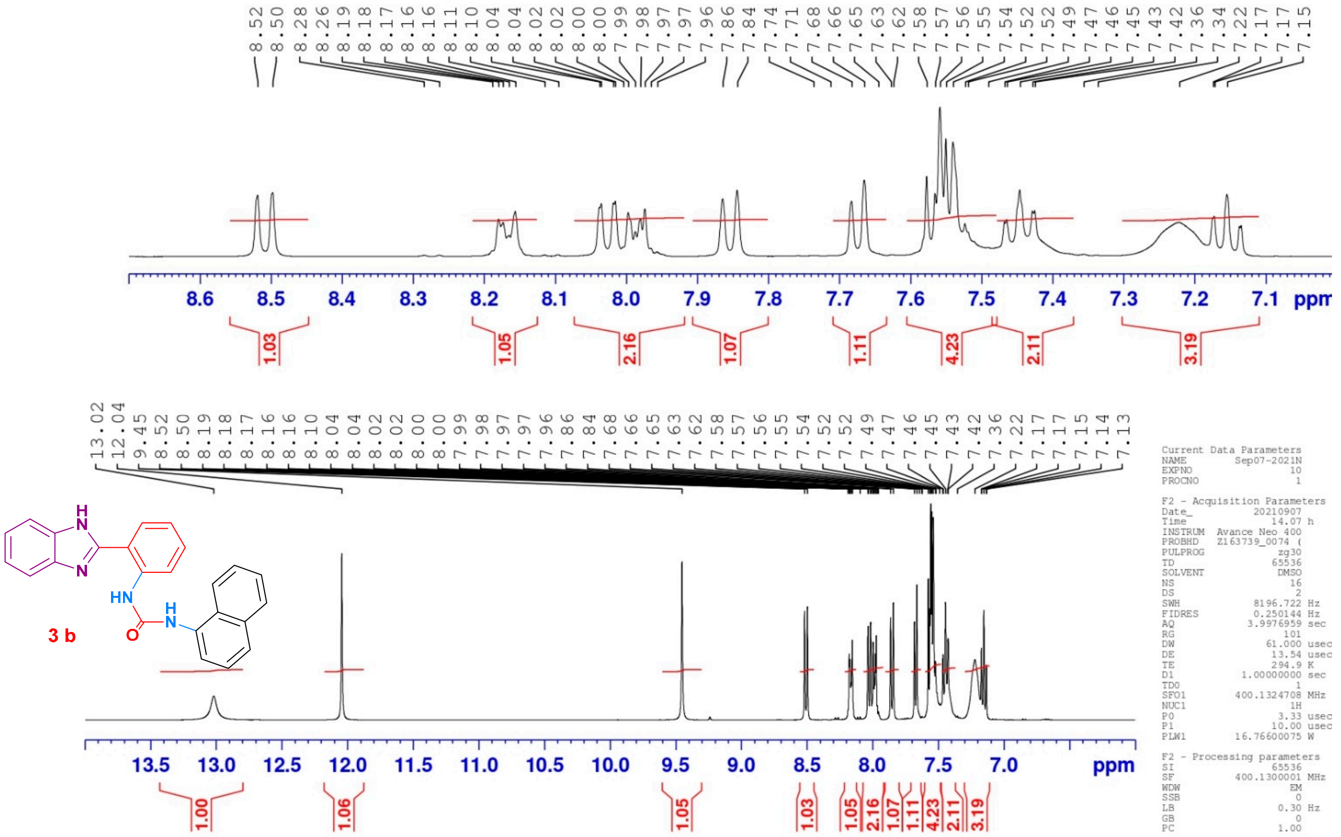

Figure S-6: ^{1}H-NMR spectrum of compound **3b**.

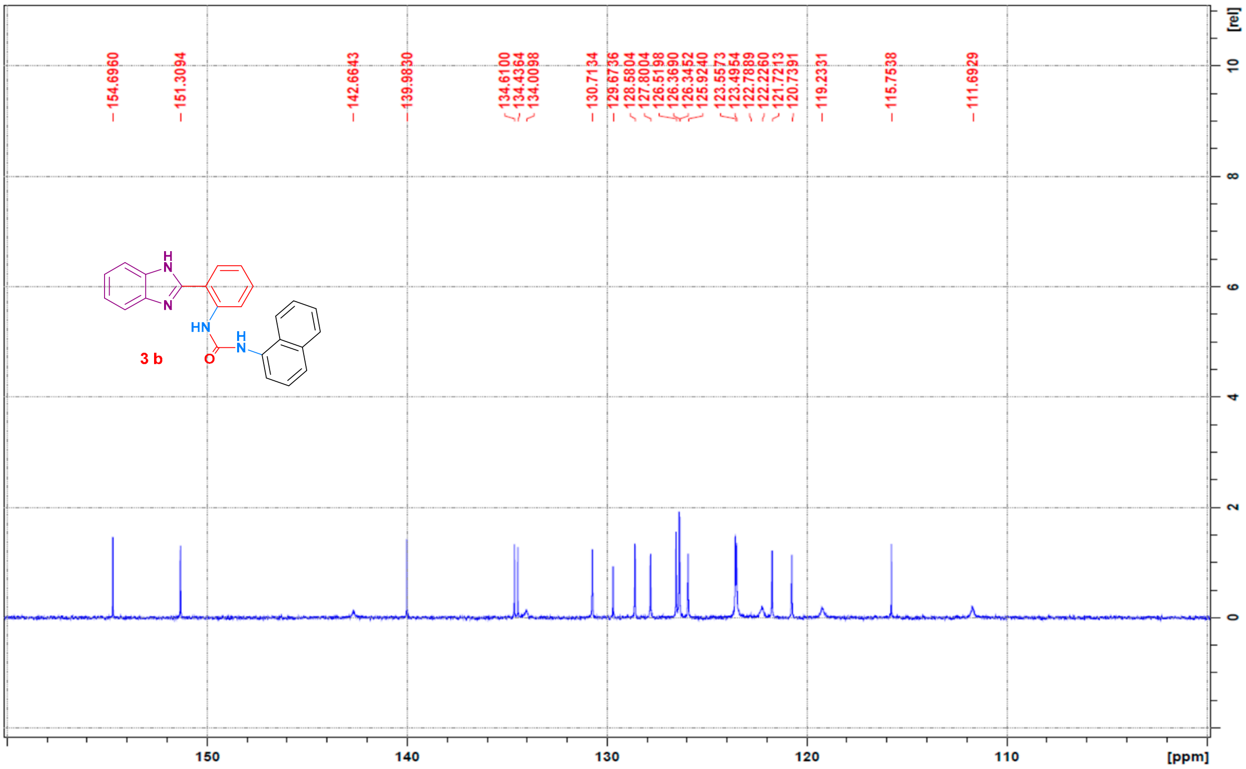

Figure S-7: ^{13}C NMR spectrum of compound **3b**.

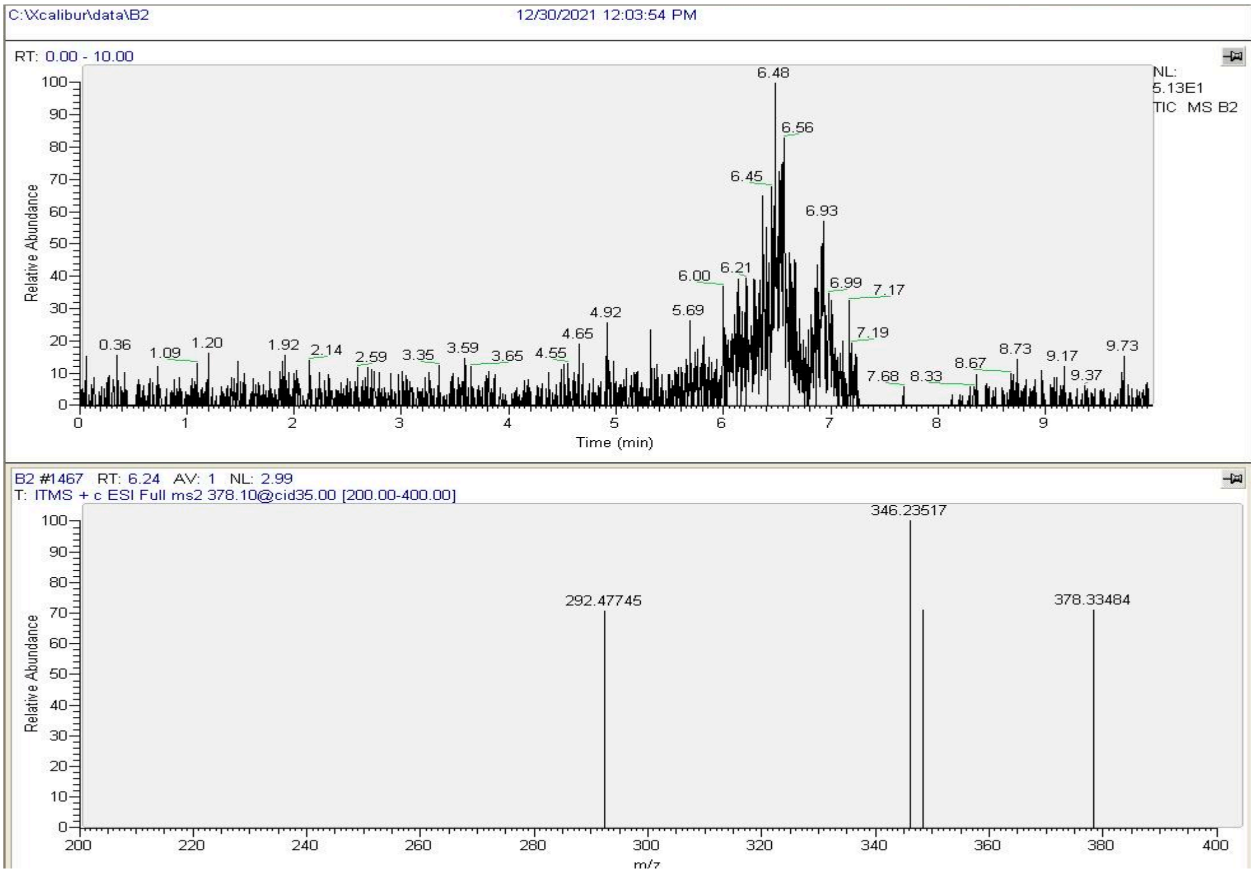

Figure S-8: Mass spectrum of compound **3b**.

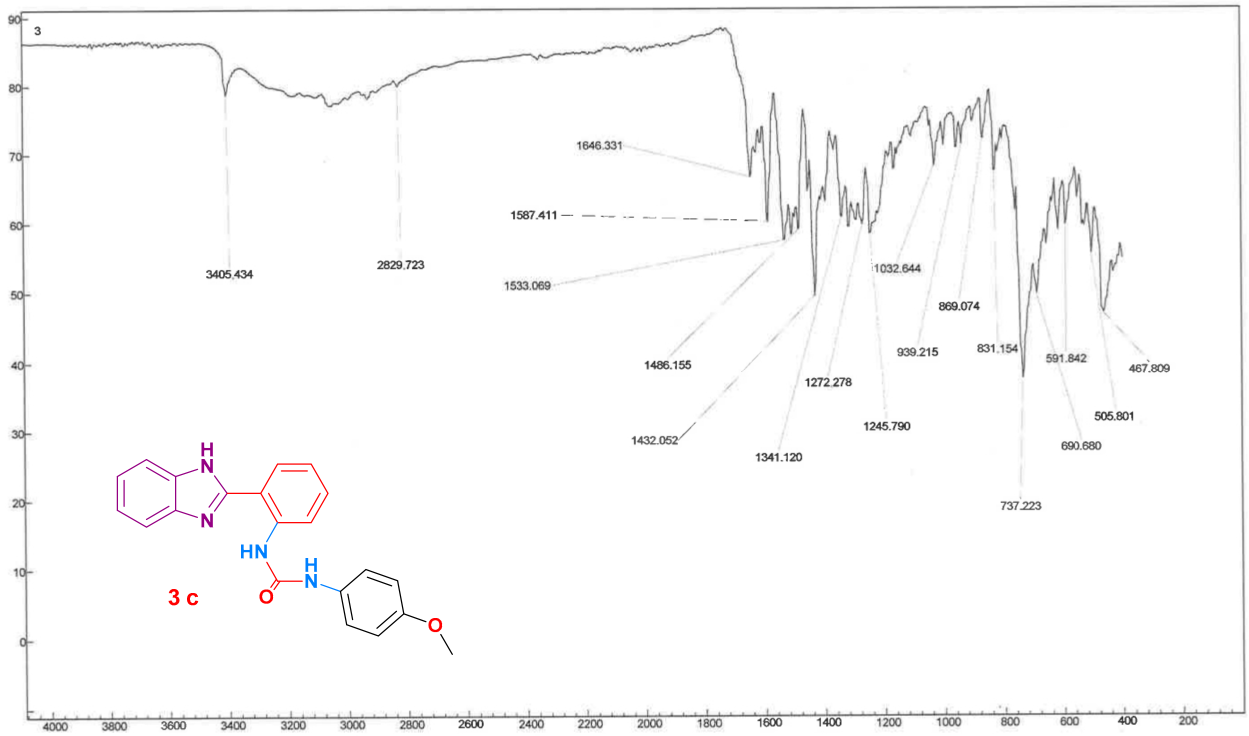

Figure S-9: FTIR spectrum of compound **3c**.

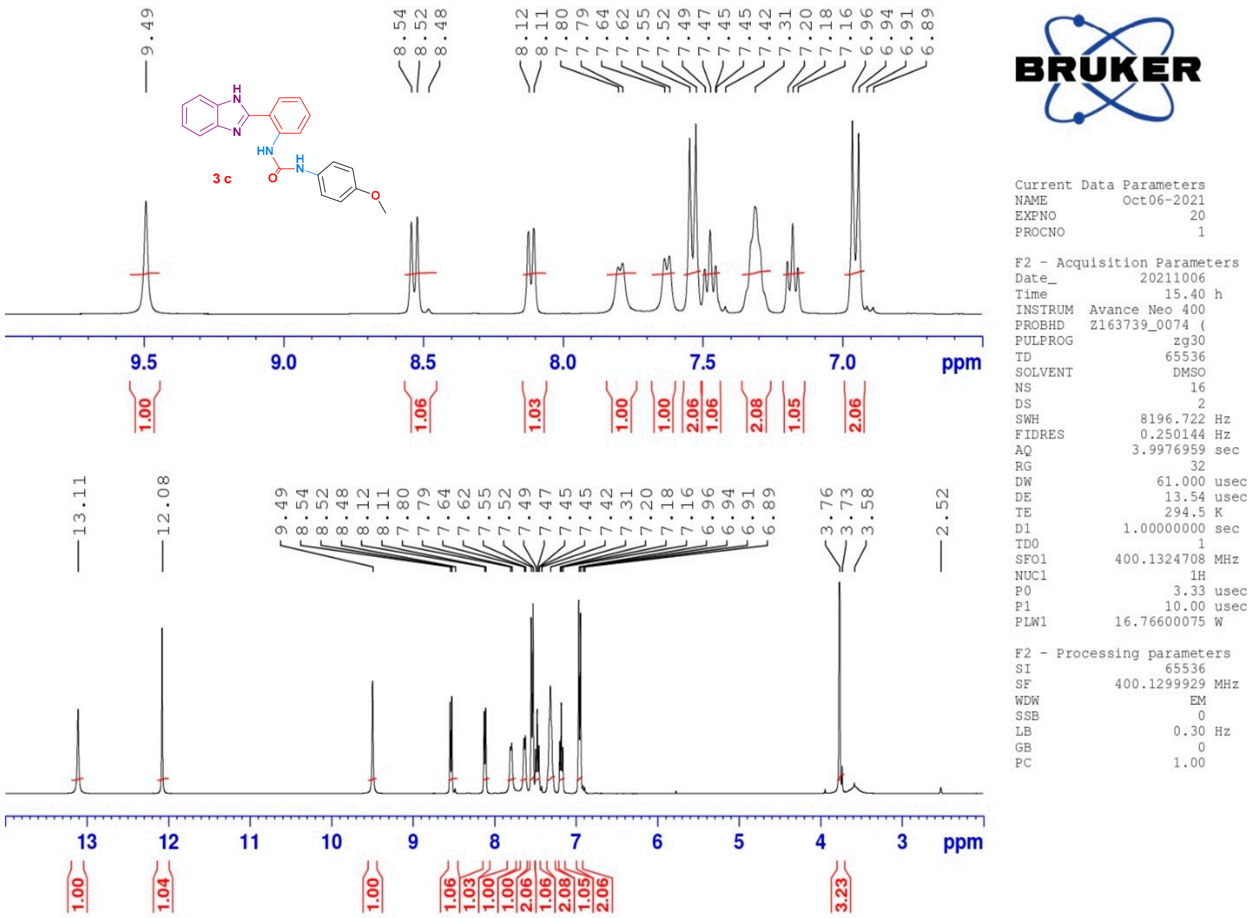

Figure S-10: ^{1}H-NMR spectrum of compound **3c**.

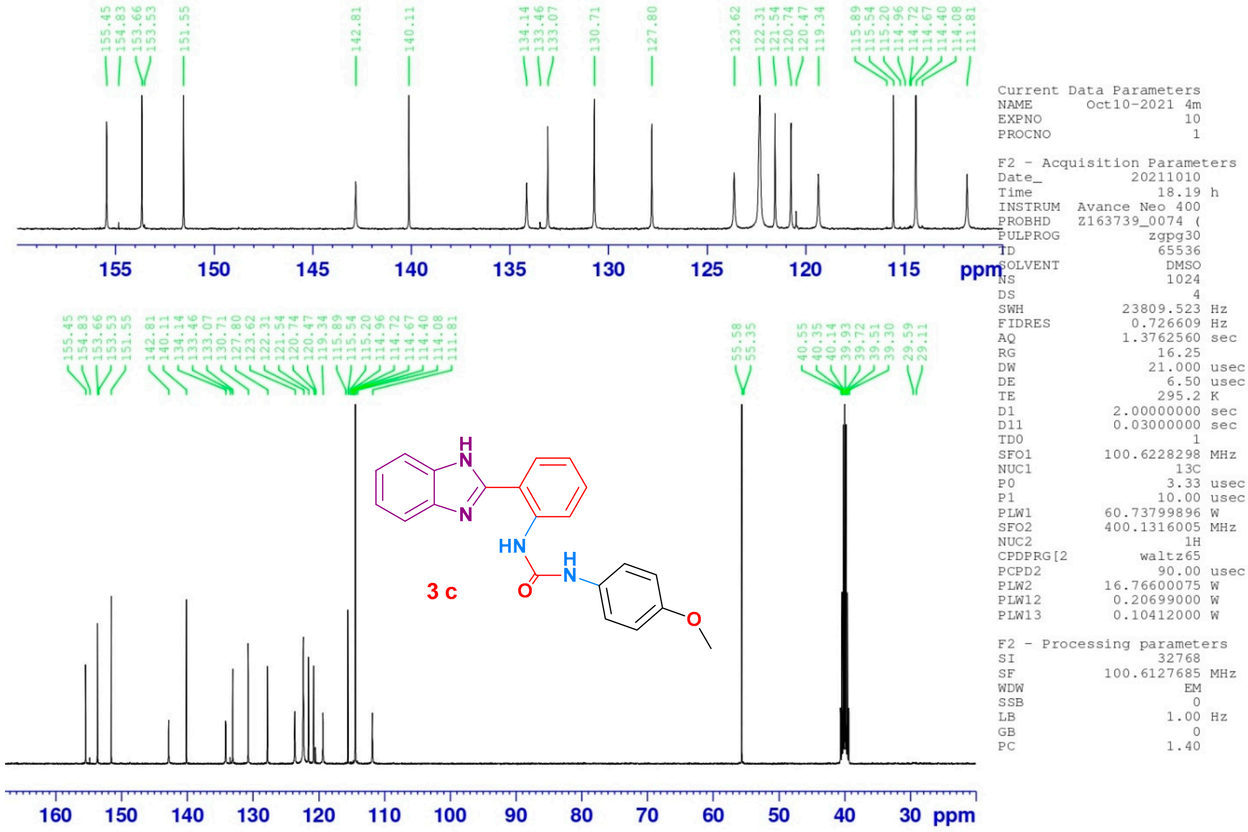

Figure S-11: ^{13}C NMR spectrum of compound **3c**.

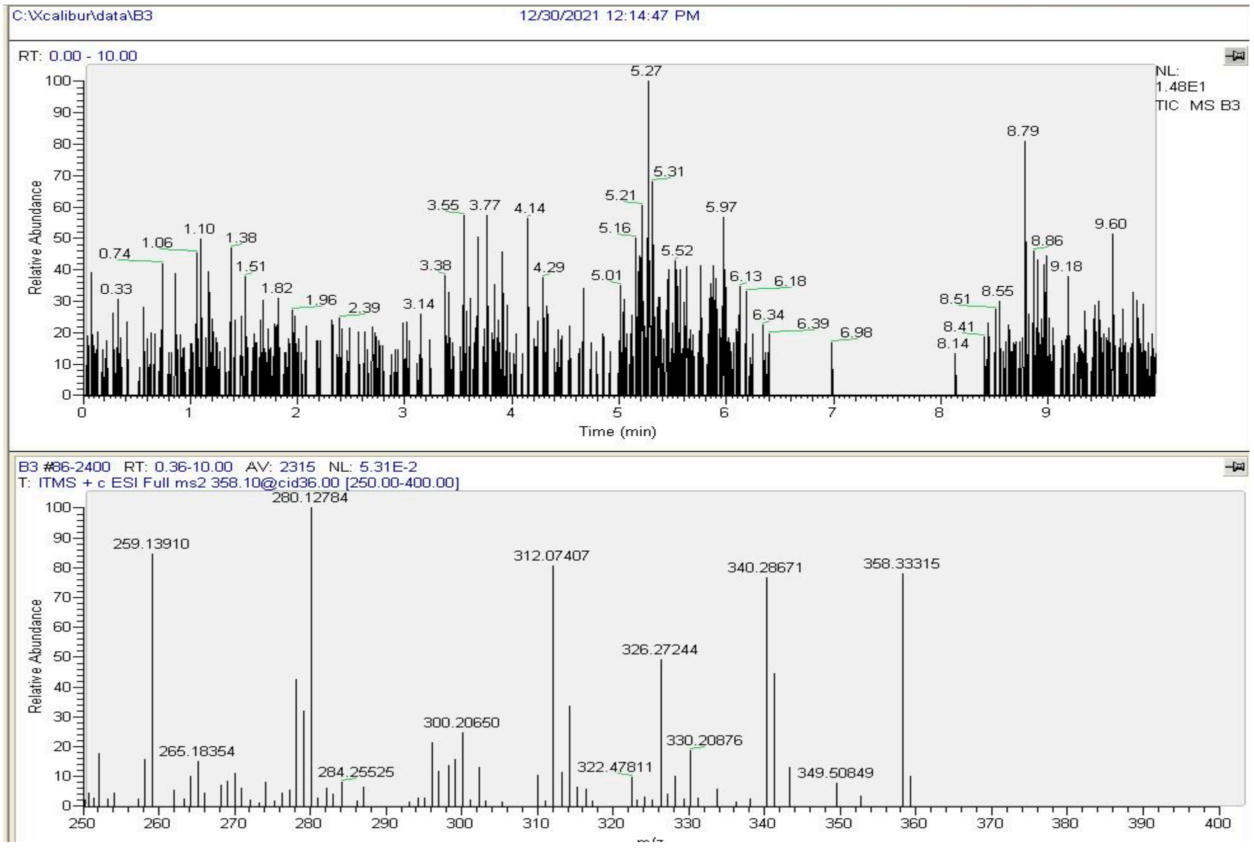

Figure S-12: Mass spectrum of compound **3c**.

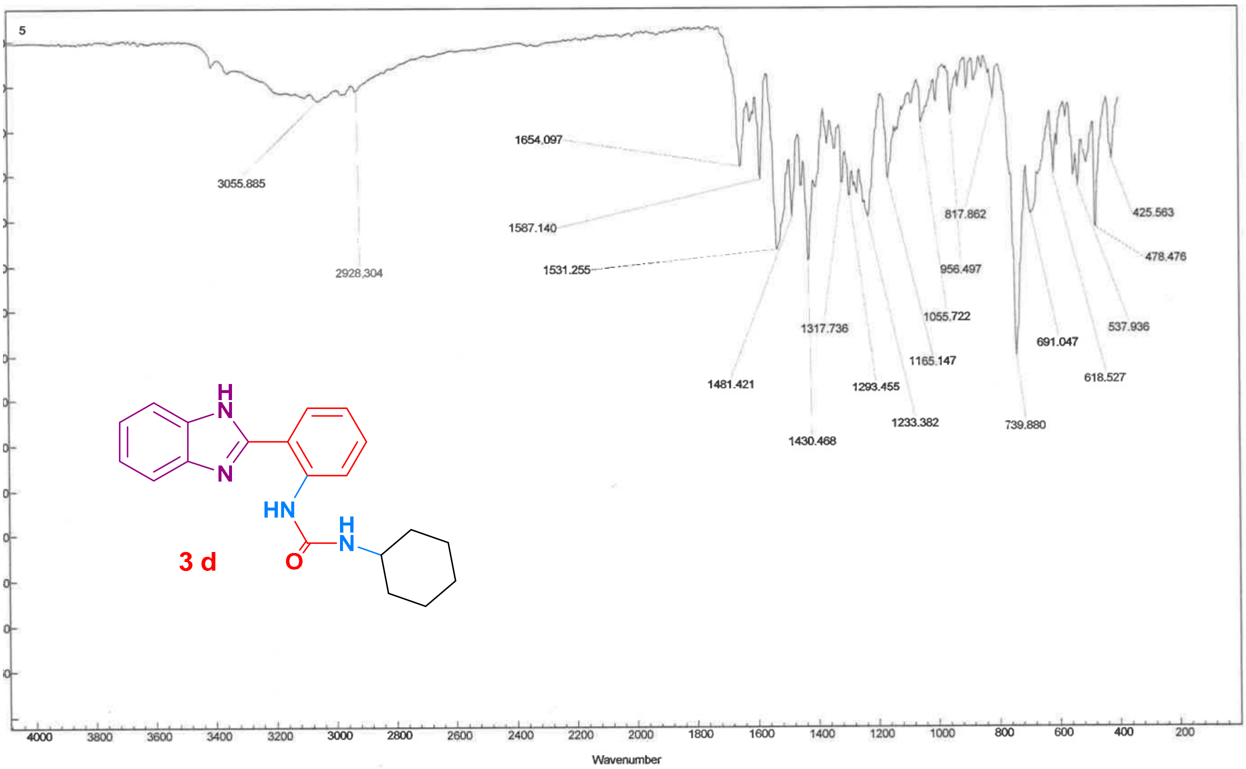

Figure S-13: FTIR spectrum of compound **3d**.

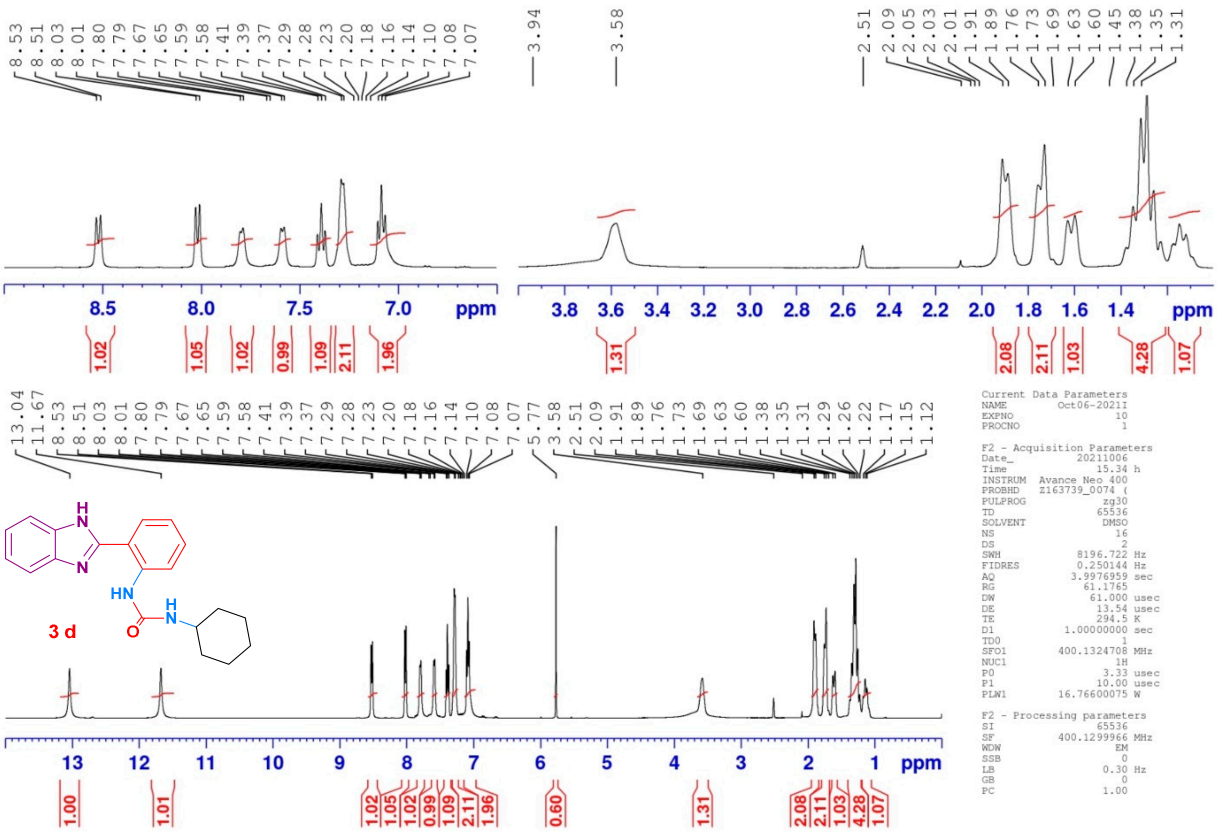

Figure S-14: ^{1}H-NMR spectrum of compound **3d**.

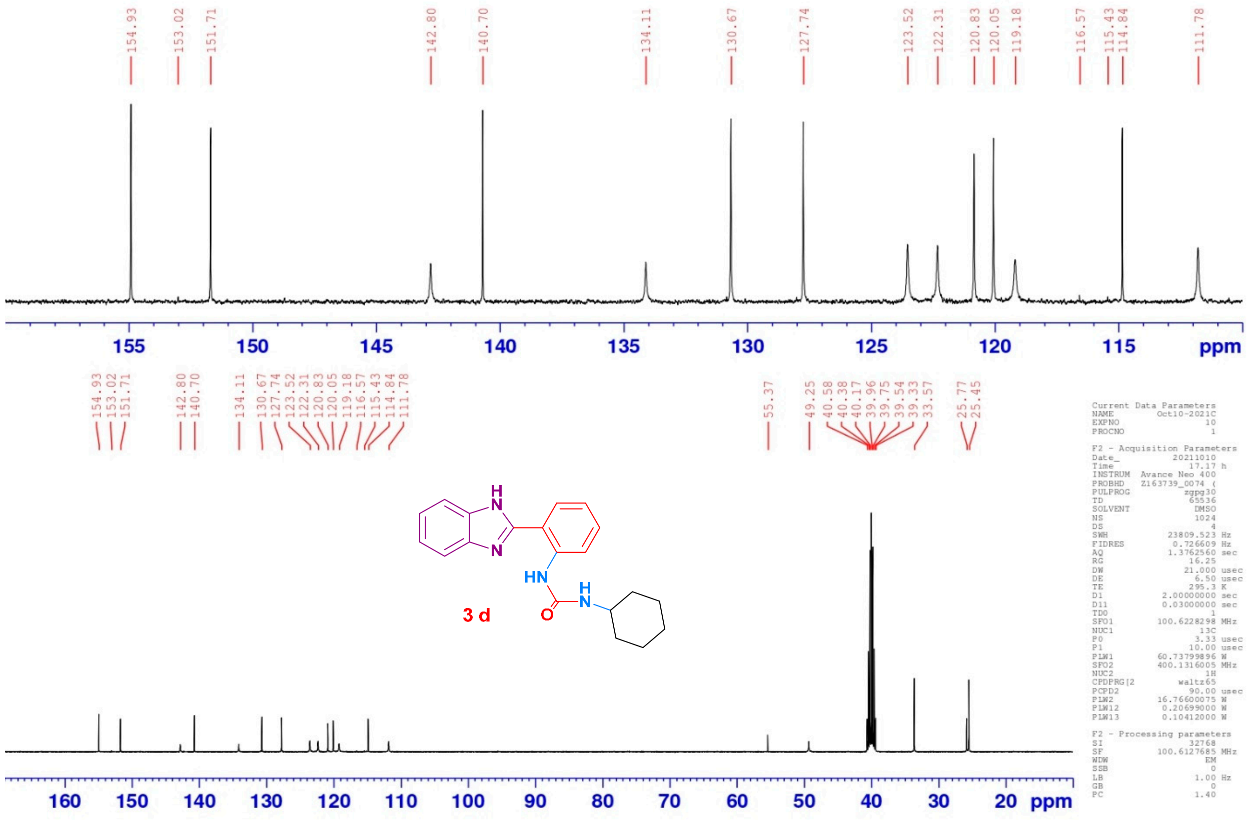

Figure S-15: ^{13}C NMR spectrum of compound **3d**.

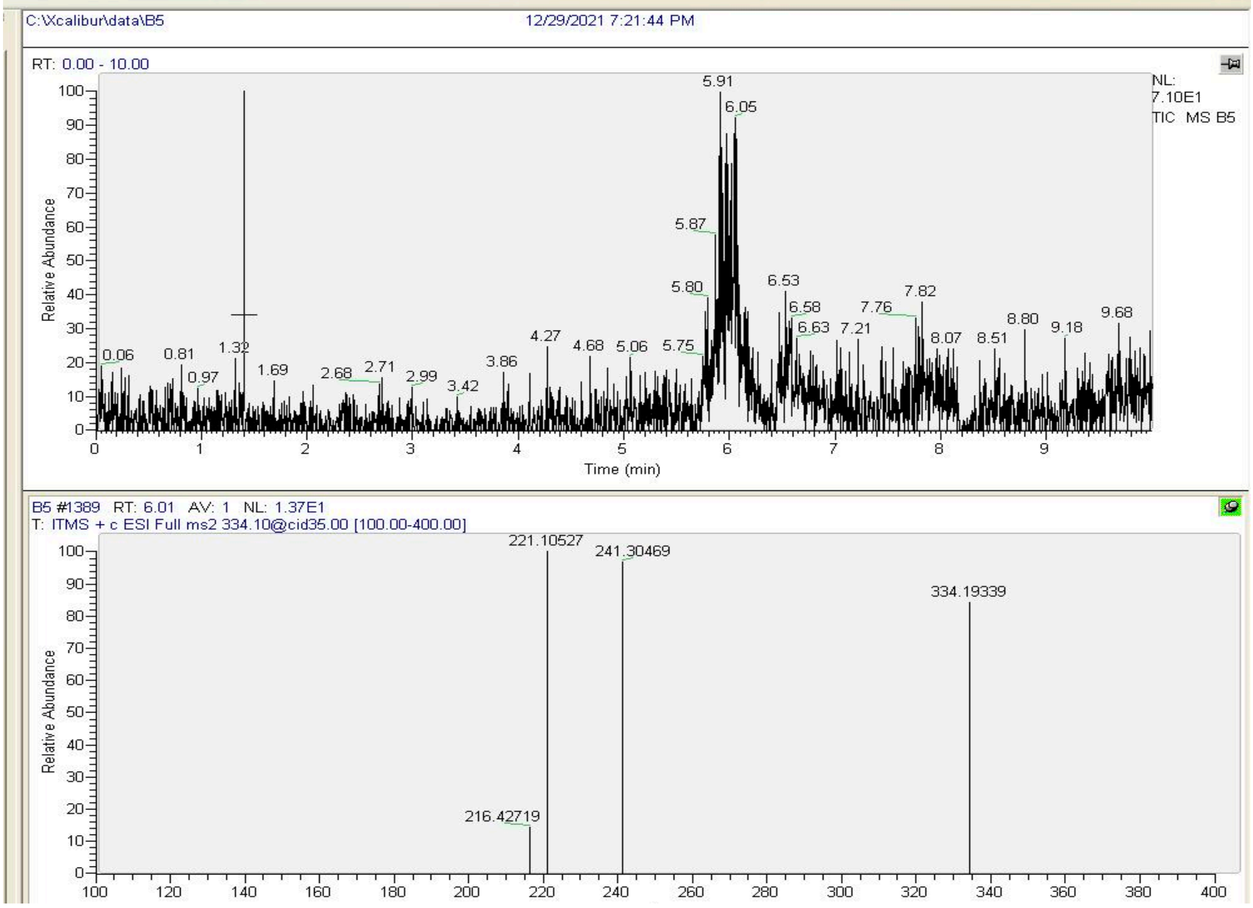

Figure S-16: Mass spectrum of compound **3d**.

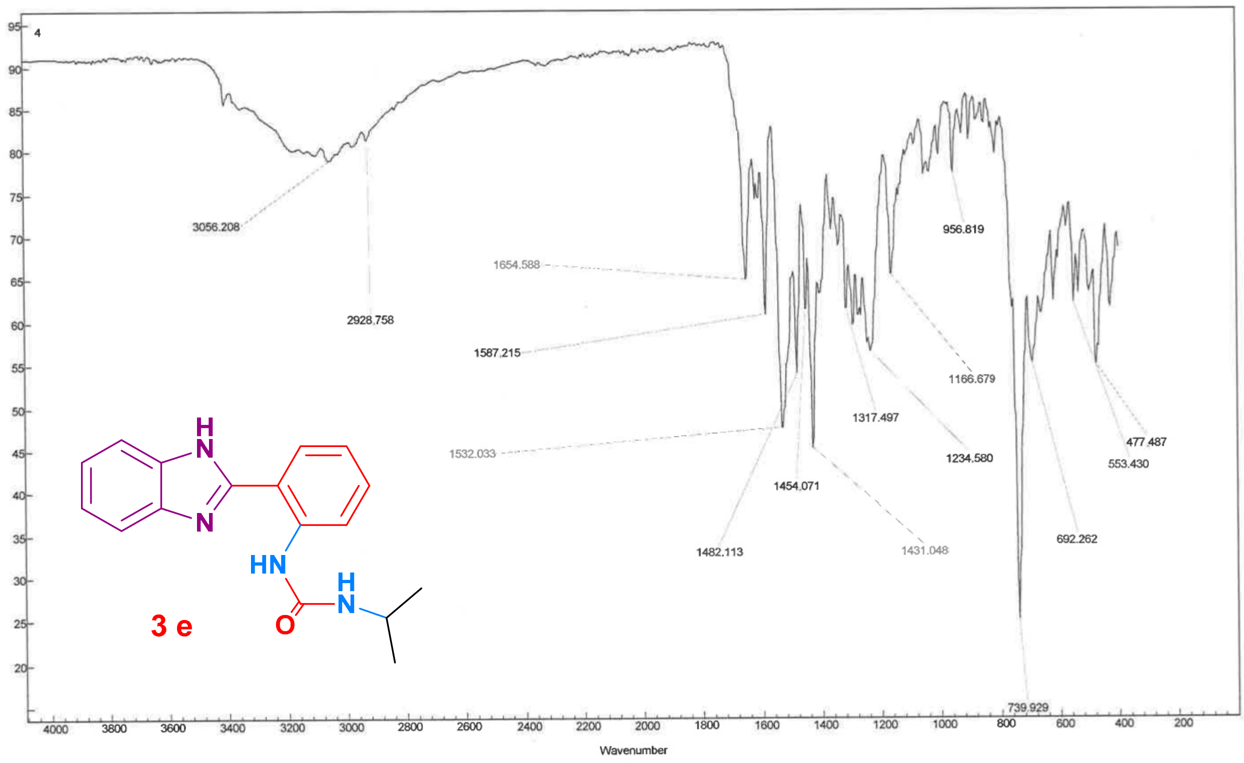

Figure S-17: FTIR spectrum of compound **3e**.

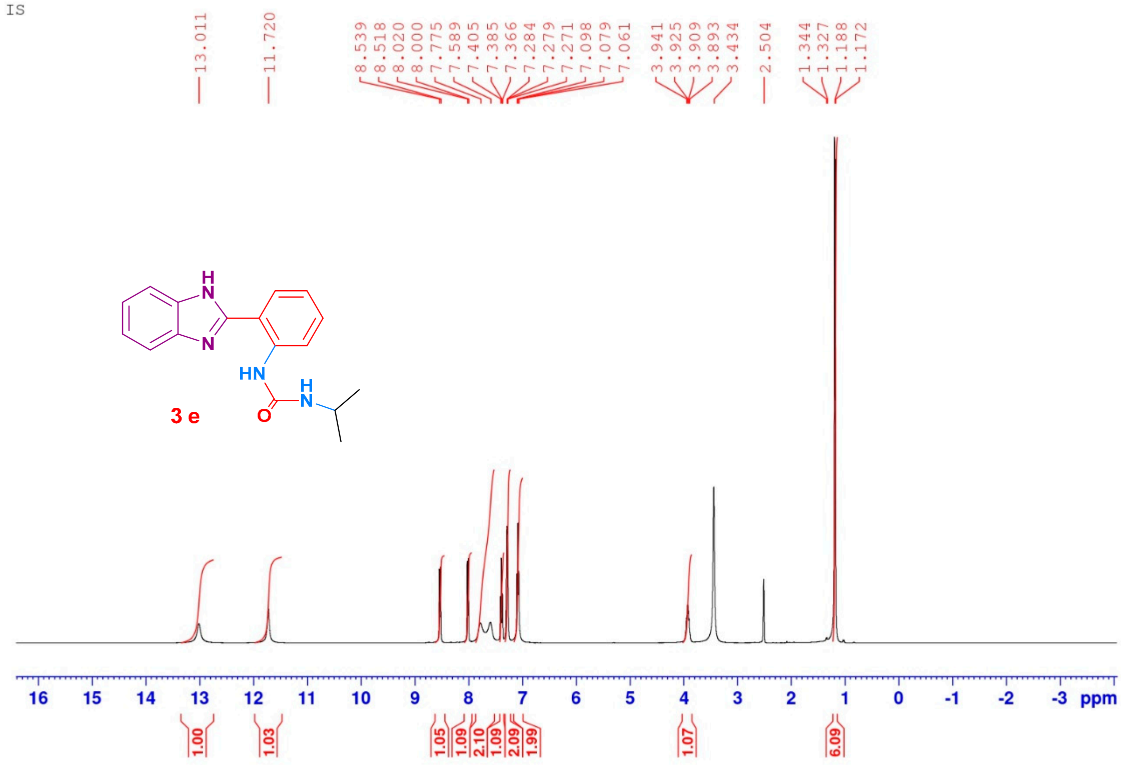

Figure S-18: ^{1}H-NMR spectrum of compound **3e**.

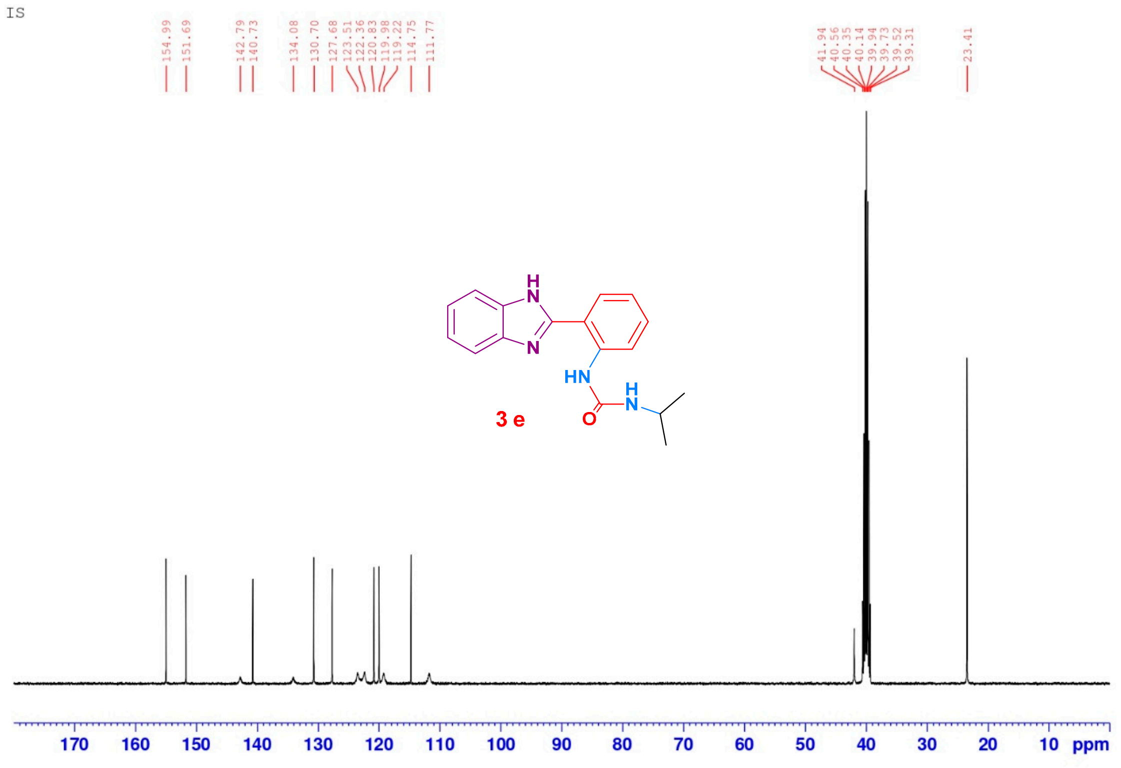

Figure S-19: ^{13}C NMR spectrum of compound **3e.**

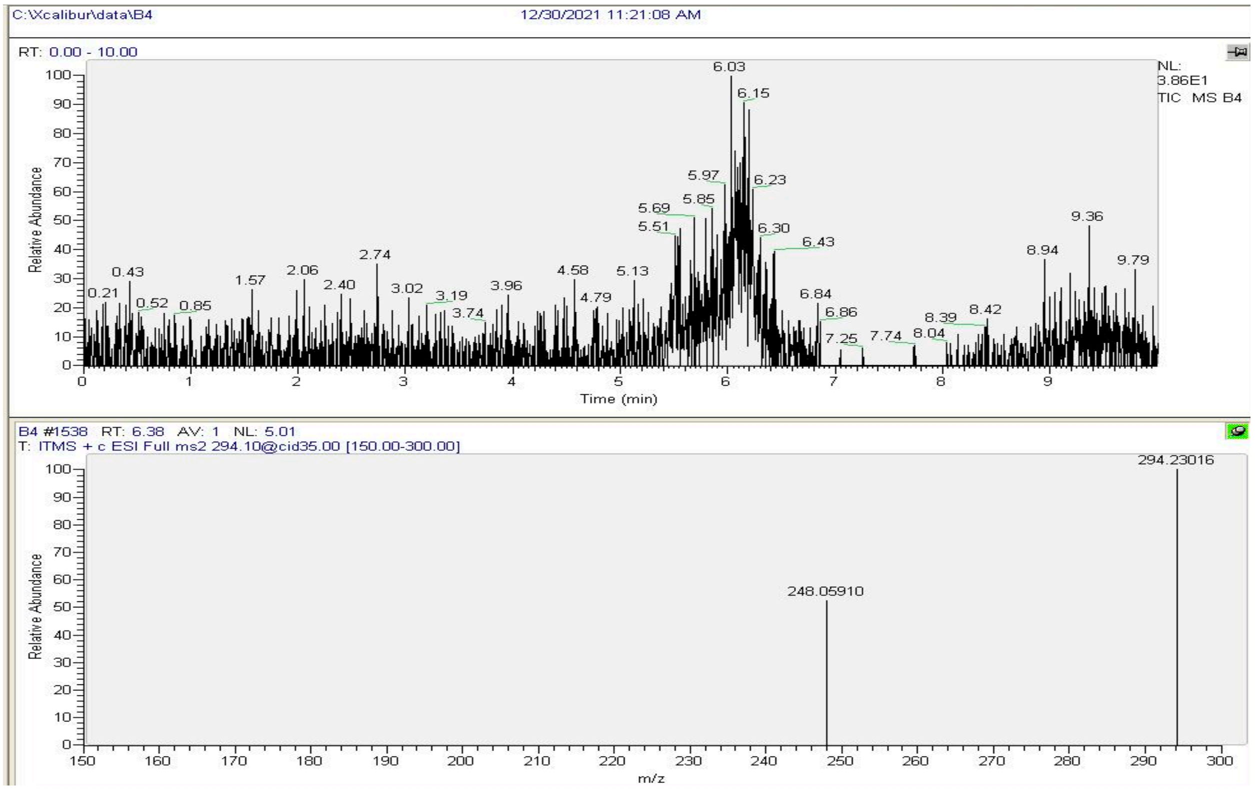

Figure S-20: Mass spectrum of compound **3e**.

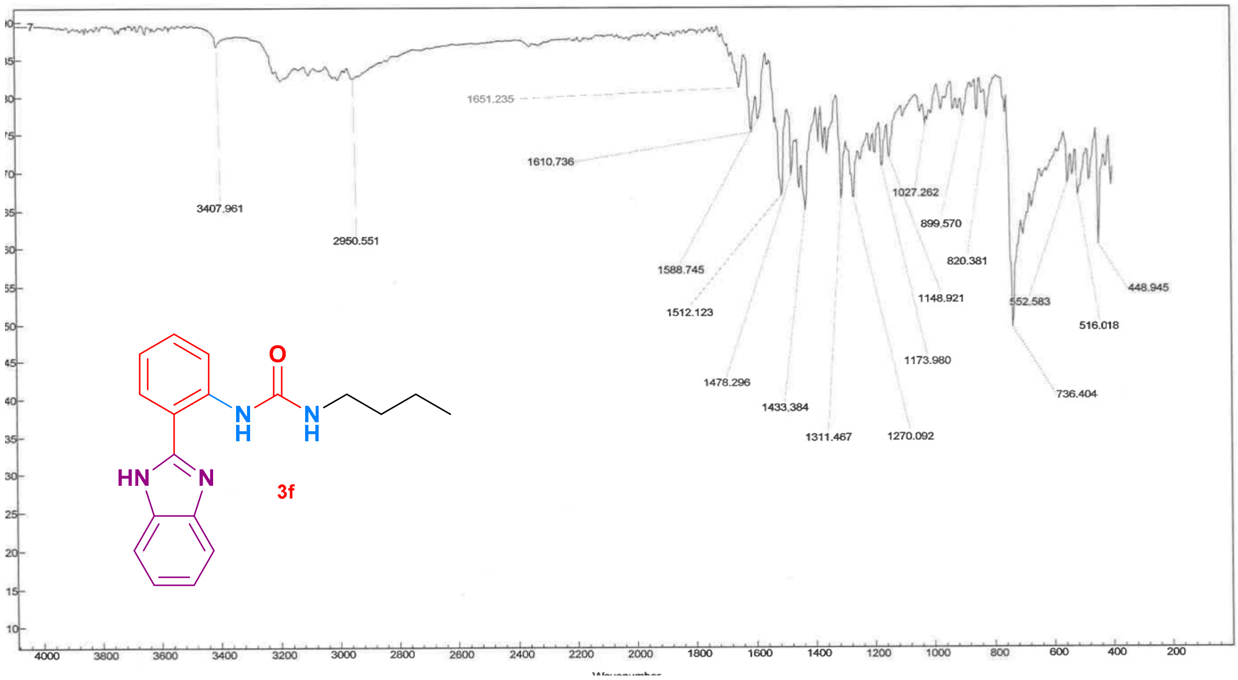

Figure S-21: FTIR spectrum of compound **3f**.

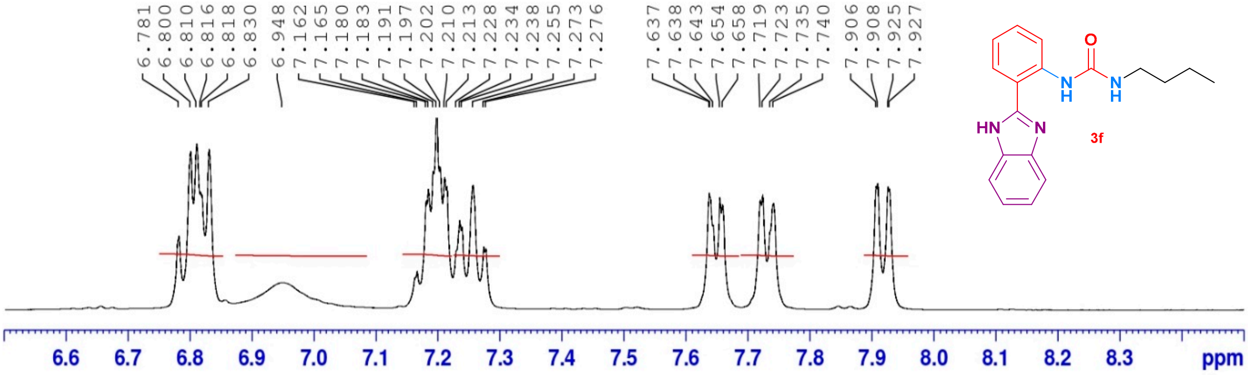

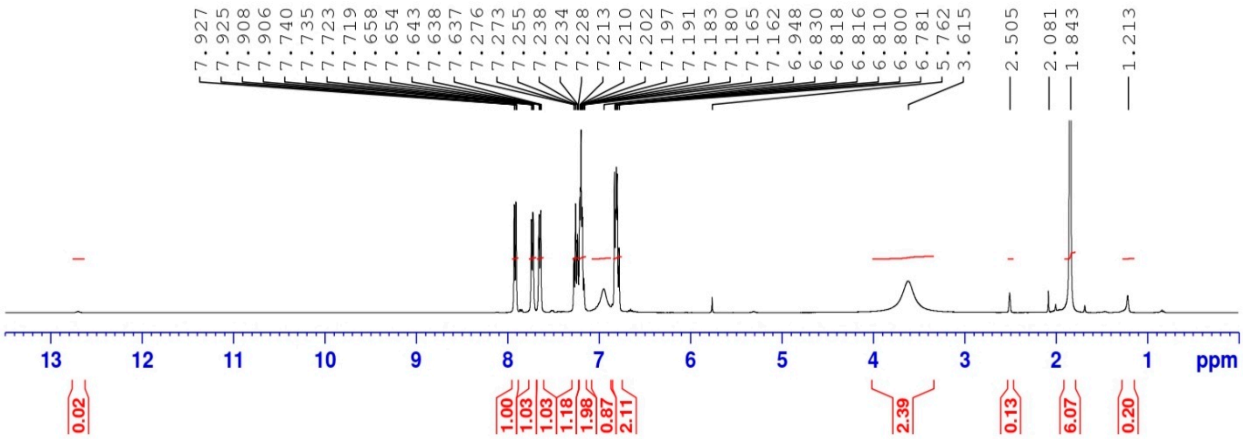

Figure S-22: ^{1}H-NMR spectrum of compound **3f**.

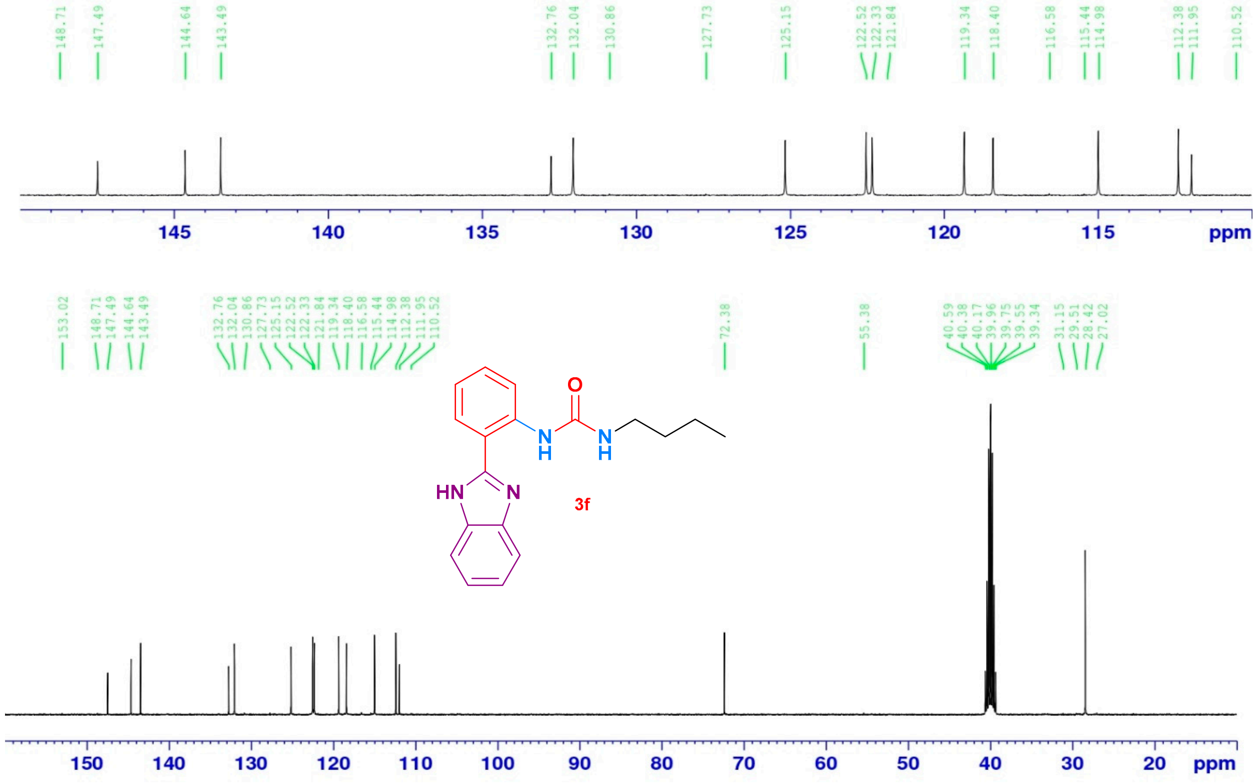

Figure S-23: ^{13}C NMR spectrum of compound **3f.**

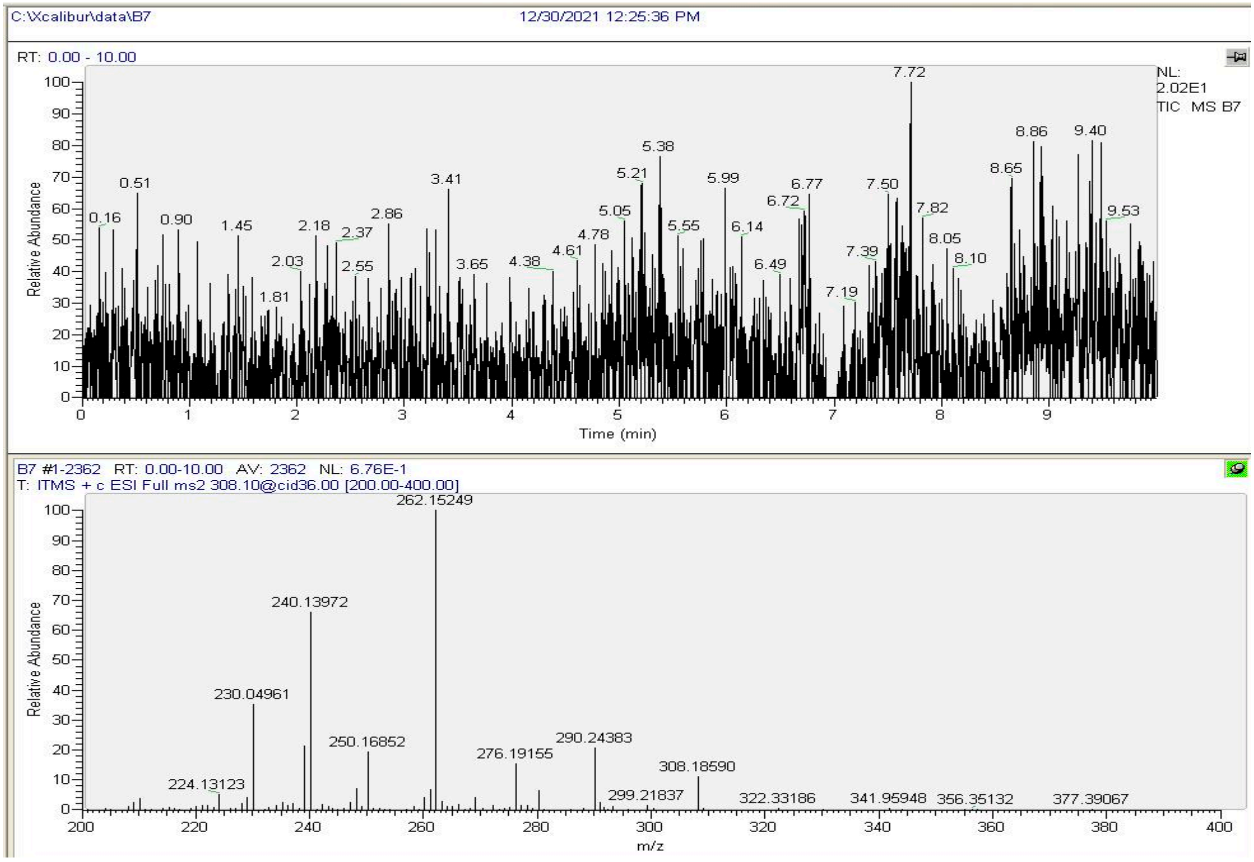

Figure S-24: Mass spectrum of compound **3f**.

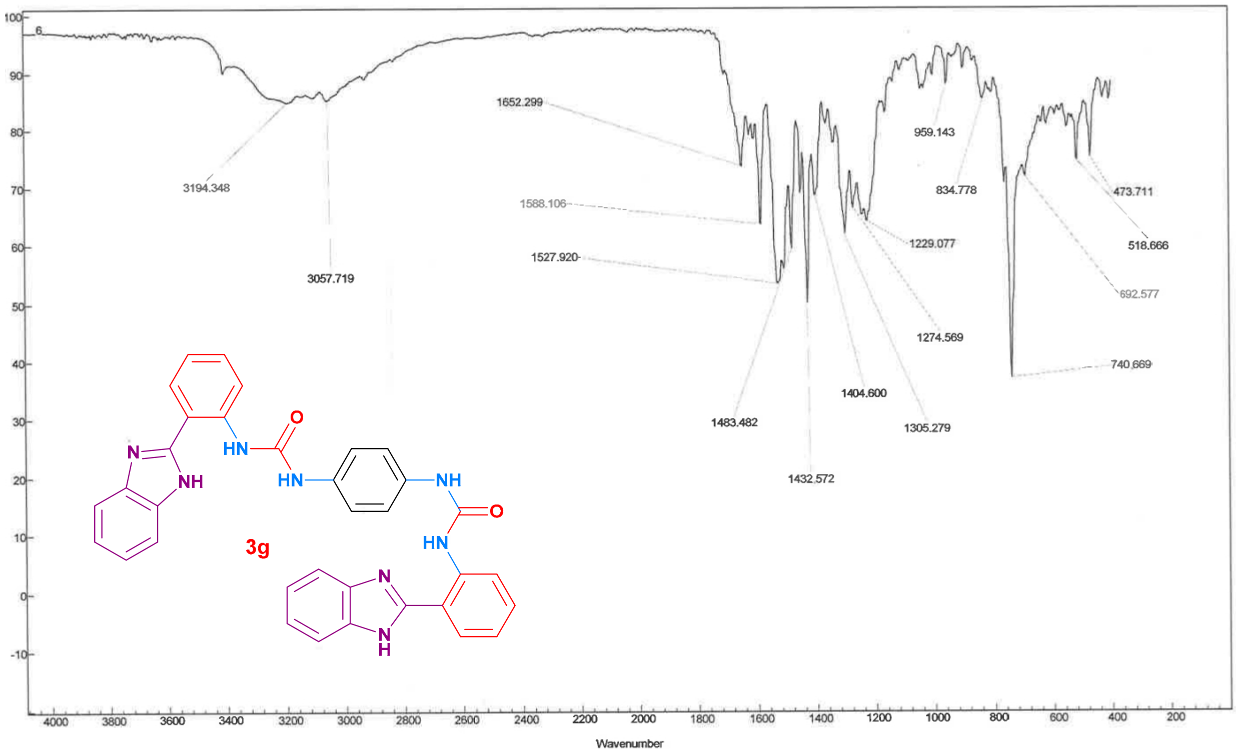

Figure S-25: FTIR spectrum of compound **3g**.

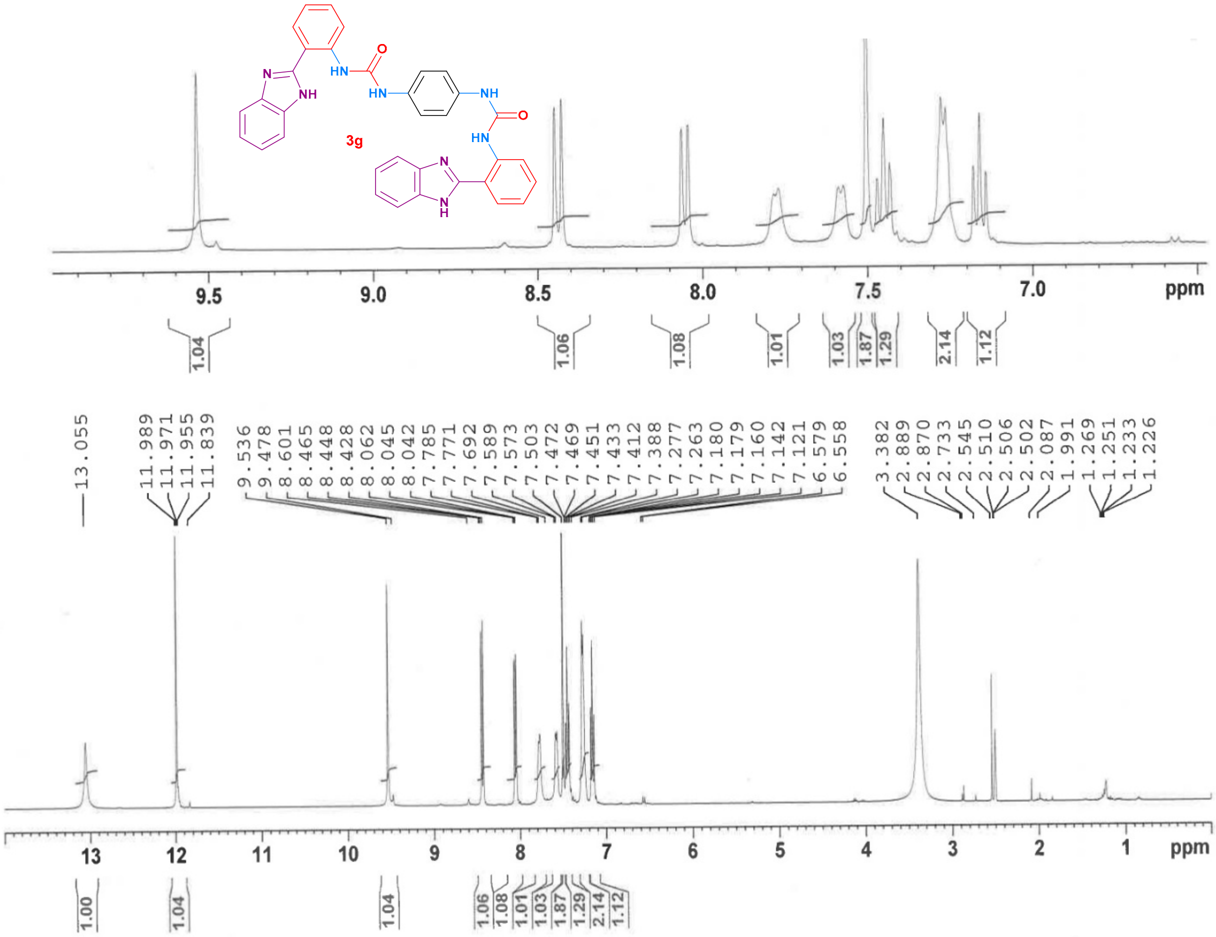

Figure S-26: ¹H-NMR spectrum of compound **3g**.

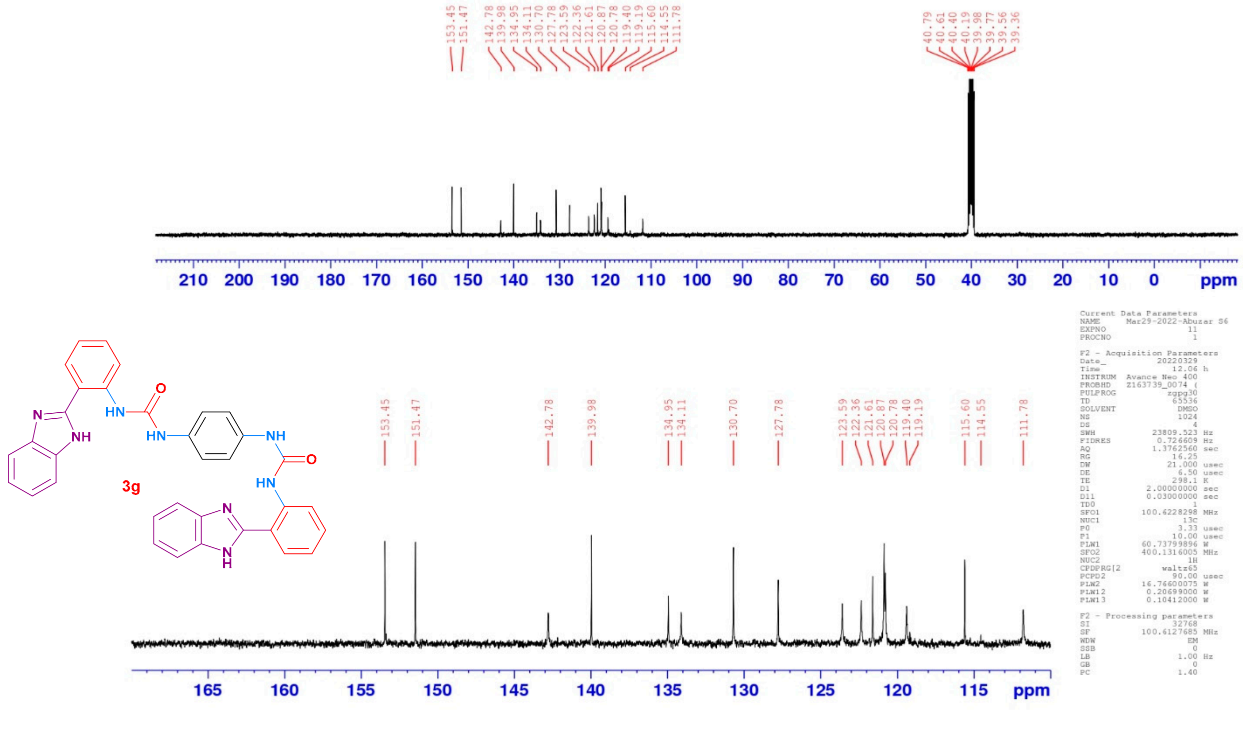

Figure S-27: ^{13}C NMR spectrum of compound **3g**.

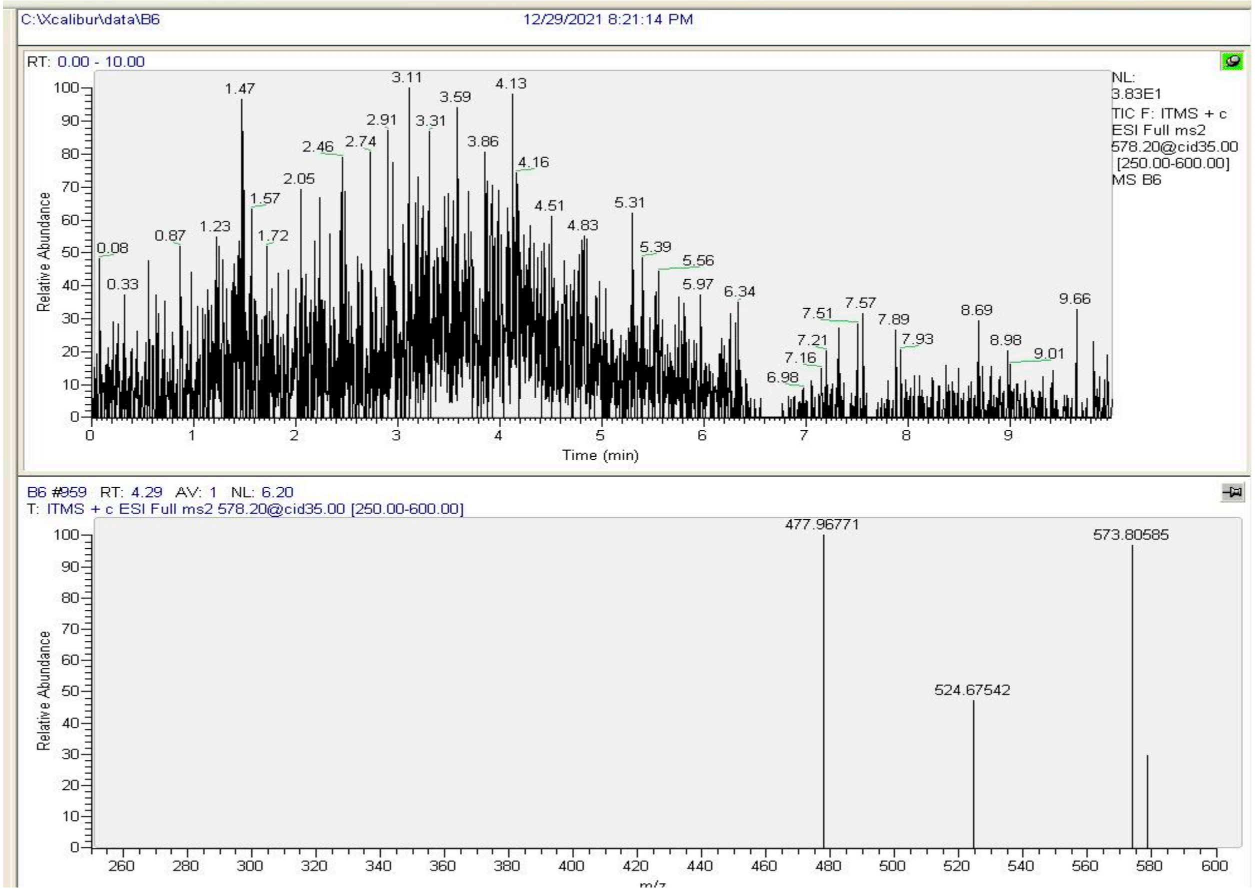

Figure S-28: Mass spectrum of compound **3g**.

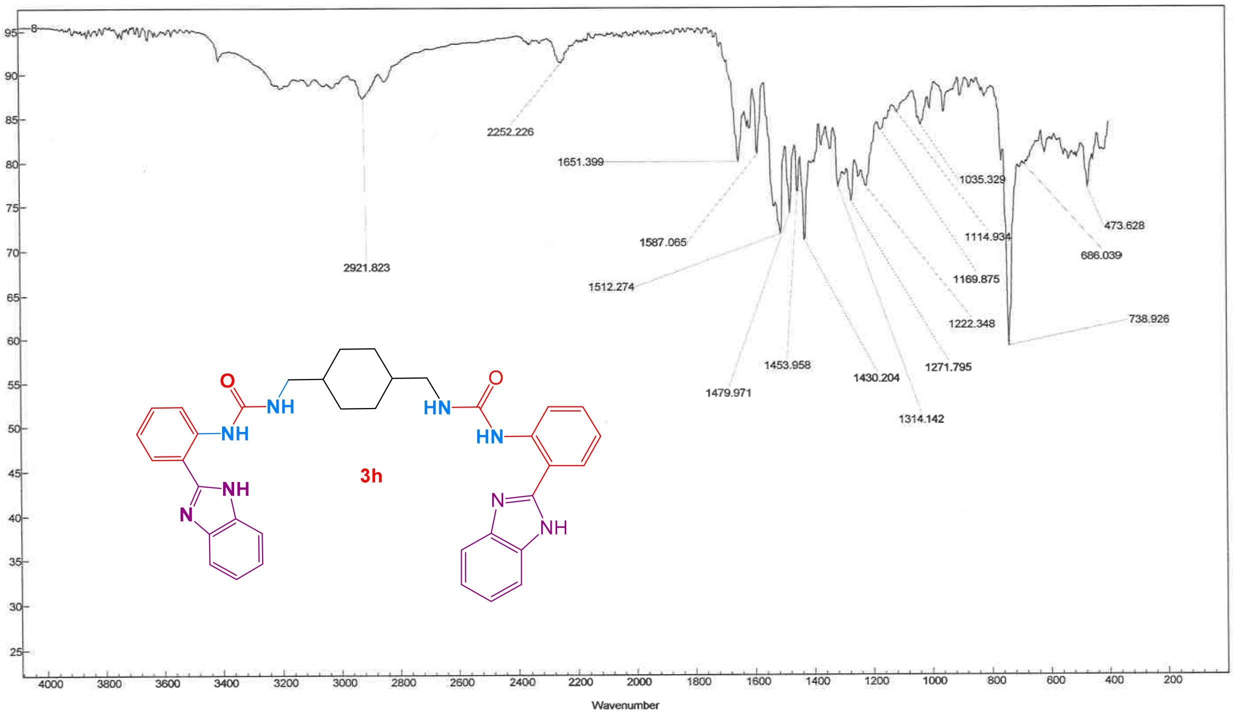

Figure S-29: FTIR spectrum of compound **3h**.

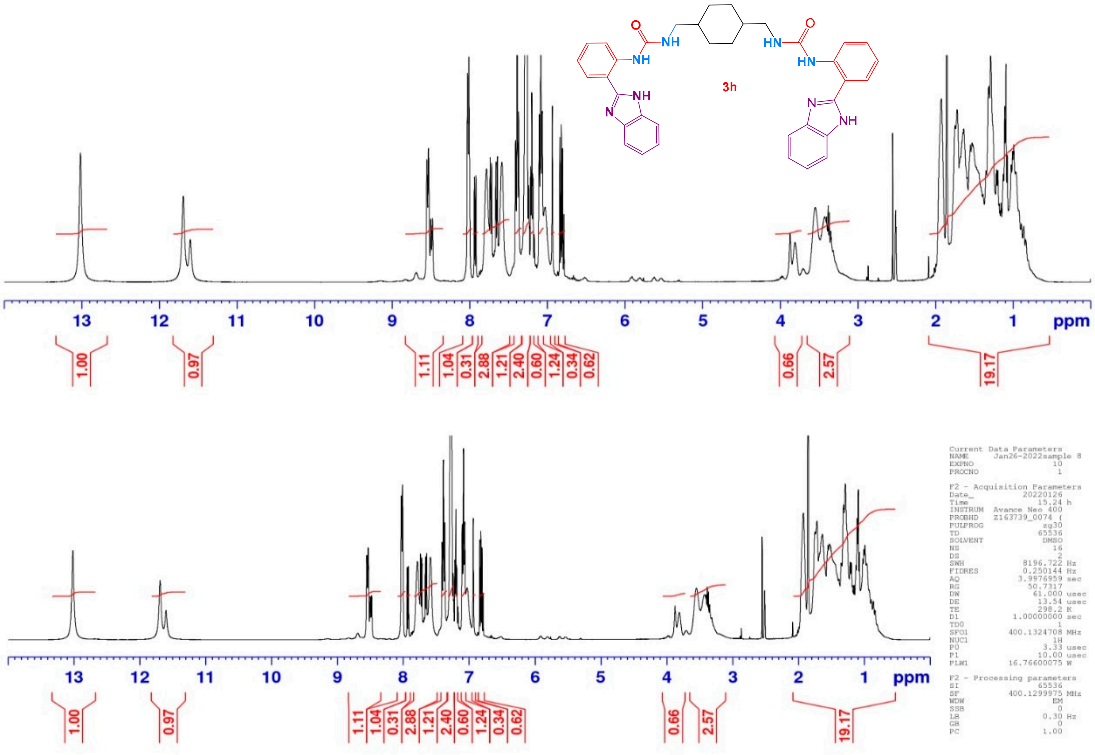

Figure S-30: ^{1}H-NMR spectrum of compound **3h**.

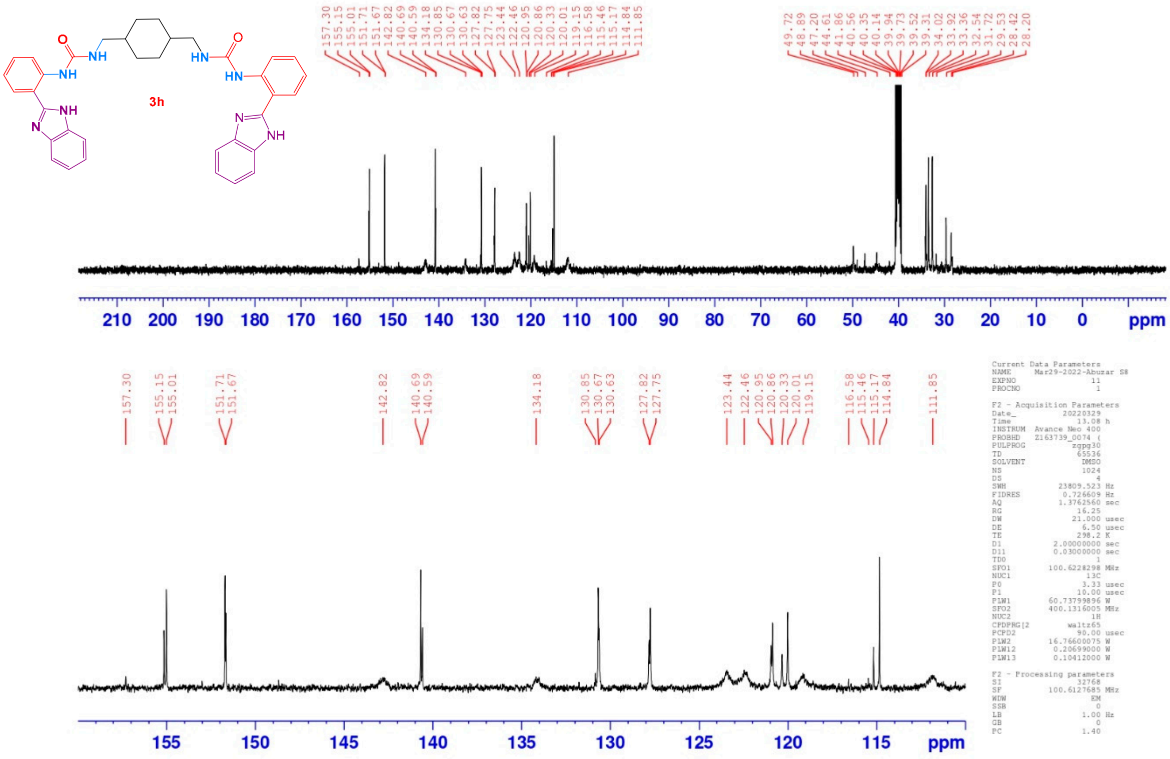

Figure S31: ^{13}C NMR spectrum of compound **3h**.

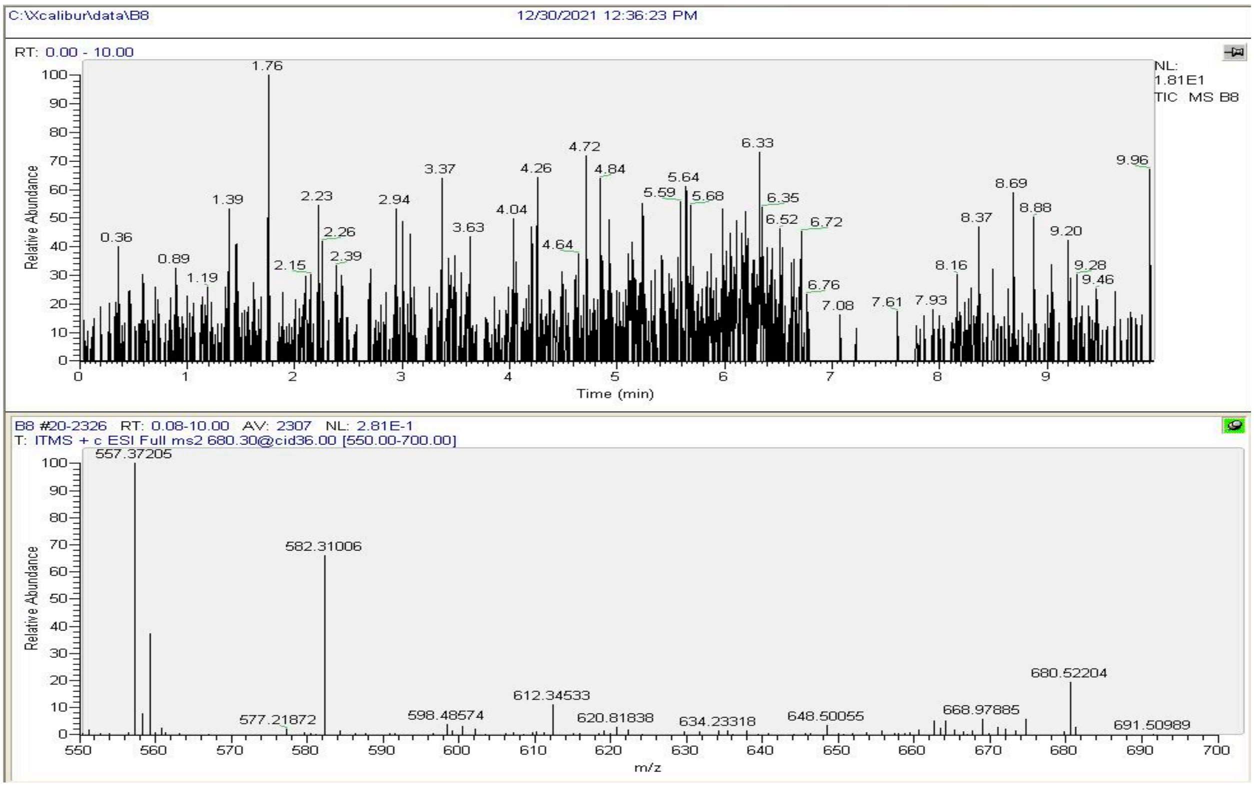

Figure S-32: Mass spectrum of compound **3h**
